# The clinical impact of prophylactic concomitant left atrial appendage occlusion during cardiac surgery: A systematic review and meta-analysis

**DOI:** 10.1016/j.ahjo.2025.100534

**Published:** 2025-03-26

**Authors:** Chengji Zhao, Evaldas Girdauskas, Jan W. Schoones, Robert J.M. Klautz, Meindert Palmen, Anton Tomšič

**Affiliations:** a Department of Cardiothoracic Surgery, Leiden University Medical Centre, Leiden, Netherlands; b Department of Cardiothoracic Surgery, Augsburg University Medical Centre, Augsburg, Germany; c Directorate of Research Policy, Leiden University Medical Centre, Leiden, Netherlands

**Keywords:** Cardiac surgery, Left atrial appendage, Thromboembolic complication

## Abstract

**Background:**

Recently, concomitant left atrial appendage occlusion (LAAO) has emerged as prophylactic treatment option for preventing thromboembolic events in patients undergoing cardiac surgery with no known history of atrial fibrillation. The efficacy of prophylactic LAAO remains unknown.

**Methods:**

PubMed, Embase, Web of Science, Emcare, and the Cochrane Library were searched for studies on prophylactic LAAO in patients undergoing cardiac surgery. The primary endpoints were postoperative thromboembolic complications and postoperative atrial fibrillation (POAF).

**Results:**

Three randomized trials and seven retrospective observational studies were included: in total, 7369 patients received either prophylactic LAAO (*n* = 3823) or no prophylactic LAAO (*n* = 3546) during their index cardiac surgery. Prophylactic LAAO reduced the risk of early thromboembolic events by 58 % (risk ratio: 0.42; 95 % confidence interval: 0.25 to 0.73; *p* = 0.002; I^2^ = 0 %) with an estimated absolute risk reduction of 0.8 %. On the other hand, a higher risk, albeit statistically not significant, of POAF was seen with LAAO (risk ratio: 1.15; 95 % confidence interval: 1.00 to 1.32; *p* = 0.051; I^2^ = 64 %). Prophylactic LAAO also reduced the risk of all-time thromboembolic complications by 52 % (hazards ratio: 0.48; 95 % CI: 0.29 to 0.80; *p* = 0.005; I^2^ = 41 %).

**Conclusion:**

Prophylactic LAAO was associated with a reduction in early and all-time thromboembolic events but demonstrated a potential relation to a higher risk of POAF.

## 1 Introduction

Post-operative atrial fibrillation (POAF) is a common complication in patients undergoing cardiac surgery. It is estimated that up to 40 % of these patients develop POAF that is in turn associated with a prolonged hospital stay, risk of heart failure or rehospitalization, and an increased risk of post-operative thromboembolic events [[1], [2], [3], [4]]. POAF is also increasingly being recognized as a marker of poorer prognosis, with an increased risk of developing late atrial fibrillation (AF), and hereto related morbidity and mortality [5,6].

In patients with known AF, surgical ablation can effectively restore sinus rhythm while left atrial appendage occlusion (LAAO) is effective at preventing postoperative thromboembolic complications [[7], [8], [9]]. The detrimental effect of POAF in patients without known preoperative history of AF has generated interest in performing LAAO as preventive concomitant procedure to prevent postoperative thromboembolic complications. Studies performed to date have shown conflicting results, with some studies supporting the efficacy of prophylactic LAAO and others not only failing to support its efficacy but actually demonstrating a negative clinical effect [10,11]. These unexpected findings might be related to the biological function of the left atrial appendage and the pathophysiology of POAF, that might differ to the pathophysiology of AF in other settings.

We conducted a systematic review and meta-analysis to evaluate the impact of routine LAAO in patients undergoing cardiac surgery. In particular, our aim was to determine the efficacy and safety of concomitant LAAO to prevent thromboembolic complications in patients undergoing cardiac surgery without a prior history of AF.

## 2 Methods

This systematic review and meta-analysis were conducted according to the Preferred Reporting Items for Systematic reviews and Meta-Analyses (PRISMA) 2020 statement [12].

### 2.1 Search strategy

A literature search of PubMed, Embase (OVID version), Web of Science, Emcare (OVID version), and the Cochrane Library was performed by a biomedical information specialist (J.W.S.). The final search was conducted on September 19th 2024 and the search strategy is elaborated in Supplementary Material 1. After removal of duplicates, two reviewers (A.T. and C.Z.) independently assessed the titles and abstracts of the remaining articles for eligibility. Full-text articles were assessed when this was inconclusive. Discrepancies were discussed and resolved by the third review author (M.P.). No approval of the ethical committee was needed due to the nature of this study.

### 2.2 Inclusion criteria

The following inclusion criteria were applied: patients had undergone cardiac surgery; patients were stratified into LAAO and no-LAAO groups; only patients without known history of AF were included unless when <20 % of patients in both groups presented with a history of AF without significant differences in the prevalence of AF in both groups or the authors conducted a sub-analysis on patients without pre-operative AF. Left atrial appendage occlusion (LAAO) was defined as surgical exclusion of the left atrial appendage (LAA) from the systemic circulation using a clip device, surgical amputation of the LAA and/or closure of the LAA with double layer sutures.

Only full-text articles in English that were published in peer-reviewed journals were eligible for inclusion. Other full-text articles relevant to the topic were found by searching the reference lists of the included studies.

### 2.3 Data extraction

Data extraction was independently performed by two reviewers (A.T. and C.Z.) using Microsoft Office Excel (Microsoft, Redmond, WA, USA). Data on first author, year of publication, country of origin, number of patients included, baseline patient characteristics and comorbidities, intraoperative details, and early and late postoperative mortality and morbidity were collected. Whenever studies reported propensity score matched cohorts, data was extracted from the propensity score matched groups if the data on the outcomes of interest were available. For randomized controlled trials, endpoints from the intention to treat analysis were extracted in case both intention to treat and per protocol analysis were available.

### 2.4 Risk of bias assessment

Using the ROBINS-I risk of bias tool for non-randomized trials and the RoB 2 risk of bias tool for randomized trials, two independent reviewers (A.T. and C.Z.) assessed the risk of bias of the included studies [13,14]. The study characteristics were categorized into low, medium, and high risk of bias.

### 2.5 Study endpoints

Primary endpoints were early thromboembolic events, occurring within 30 days of the cardiac surgery or during hospitalisation, and POAF. Secondary endpoints were thromboembolic events occurring at any time after surgery and late AF.

### 2.6 Statistical analysis

Continuous patient characteristics were pooled and are presented as means with 95 % confidence intervals (CIs). Binary patient characteristics were pooled and are presented as counts and percentages. Risk ratios with 95 % CIs were calculated to generate forest plots and to express differences for early outcomes. For time-to-event outcomes (all-time thromboembolic events), published hazard ratios (HRs) with 95 % CIs and, when not available, HRs with 95 % CIs obtained from reconstructed individual patient data (IPD) were used to generate forest plots. Random-effects models were used to pool the results. Heterogeneity was examined with the I^2^ statistics. The degree of heterogeneity was graded as low (I^2^ < 25 %), moderate (I^2^ = 25 % to 75 %), and high (I^2^ > 75 %).

Published Kaplan-Meier graphs were digitized using the DigitizeIt software to raw data coordinates (time and survival probability). Extracted data coordinates were processed to reconstruct IPD using the R package “IPDfromKM” (version 0.1.10). The reconstructed IPD from all studies were merged to create the study dataset and visualized in a Kaplan-Meier graph. A two-tailed p value of <0.05 was considered statistically significant. All analyses were performed with R Statistical Software (version 4.1.1, Foundation for Statistical Computing, Vienna, Austria).

## 3 Results

### 3.1 Study selection

**Fig. 1:**
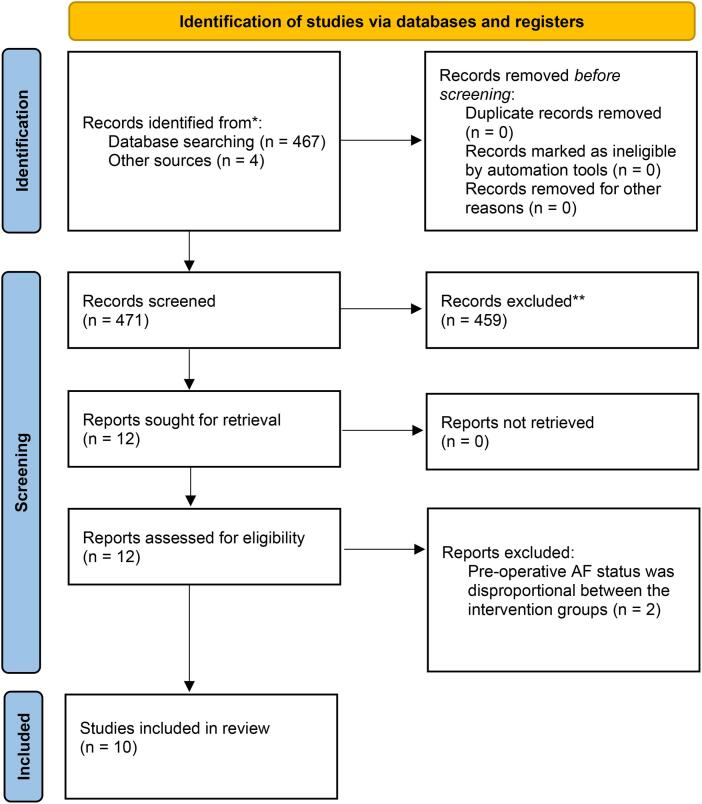
Prisma 2020 flow diagram. Two authors screened and reviewed 420 studies and 9 were included in the final review.

The search yielded 467 articles. Four other full-text articles relevant to the topic were found from other sources by screening the reference lists of the included studies. After screening the titles and abstracts, 12 articles were assessed for eligibility by full-text screening. Two additional studies were excluded, including the study by Melduni et al. in which a large proportion of patients had known history of AF, with significant differences in the treatment and control groups [15]. The final search yielded 10 individual studies published between 2009 and 2024. None of the authors published results of overlapping populations. Three of the studies were randomized controlled trials and 7 were retrospective observational studies. Six of the latter studies applied either propensity score matching or IP weighting. The detailed study flow diagram is shown in Fig. 1.

### 3.2 Prophylactic LAAO techniques

Main characteristics of the included studies are summarized in Table 1. LAAO was performed by excision or by exclusion of the LAA using sutures or stapling. Internal suture closure was used in 6 out of the 10 studies researching prophylactic LAAO. Kim et al. completed LAAO using various techniques: suture closure/amputation or closure/external closure with bioabsorbable reinforcing clips [16]. Gerdisch et al. and Noona et al. used a clip device for LAAO [17,18]. Techniques used to achieve LAAO by Yao et al. were not specified [19].

**Table 1:** Study characteristics of the included studies.

First author	Year	Country	No. of patients		Study design	LAAO technique	Postoperative OAC management	Primary and secondary Endpoints	Follow-up duration
			LAAO	No LAAO					
Ascaso et al. [10]	2022	Canada	267	267	Retrospective observational study with propensity matching	Internal surgical closure with sutures	All patients received OAC for 3 months after surgery	Early stroke, Late stroke, Late AF	LAAO group: 2.9 years (median); Control group: 5 years (median)
Chickwe et al. [11]	2023	USA	431	333	Retrospective observational study	Internal surgical closure with sutures	N/R; OAC was initiated in 32 % in the LAAO and in 12 % in the no LAAO group	Early stroke, Late stroke, POAF, Late AF	4.5 years (median)
Endo et al. [34]	2023	Japan	236.5	264.4	Retrospective observational study with IP weighting	Internal surgical closure with sutures	OAC was initiated in the presence of persistent AF or recurrent paroxysmal AF episodes	Late stroke, POAF	LAAO group: 3.6 years (mean); Control group: 3.9 years (mean)
Gerdisch et al. [17]	2022	USA	376	186	Randomized controlled trial	Clip device (AtriClip, AtriCure, Inc., Mason, OH, USA)	N/R; OAC was initiated in 24 % in the LAAO and in 16 % in the no LAAO group	Early stroke, POAF	1 year (mean)
Gerçek et al. [35]	2023	Germany	243	243	Retrospective observational study with propensity matching	Internal surgical closure with sutures	N/R	Early stroke, Late stroke, POAF	5 years (mean)
Kim et al. [16]	2013	USA	631	631	Retrospective observational study with propensity matching	Internal surgical closure with sutures or surgical amputation or external closure with bioabsorbable reinforcing clips	N/R	Early stroke, POAF	30 days/length of hospitalisation
Nagpal et al. [20]	2009	Italy	22	21	Randomized controlled trial	Internal surgical closure with sutures	N/R	Early stroke, POAF	ND
Noona et al. [18]	2024	USA	439	439	Retrospective observational study with propensity matching	Clip device	N/R; OAC was initiated in 17 % in the LAAO and in 8 % in the no LAAO group	Early stroke, POAF	30 days/length of hospitalisation
Park-Hansen et al. [21]	2018	Denmark	101	86	Randomized controlled trial	Internal surgical closure with sutures (recommended but not obligatory)	N/R; OAC was initiated in 38 % in the LAAO and in 32 % in the no LAAO group	Early stroke, Late stroke, POAF	3.7 years (mean)
Yao et al. [19]	2018	USA	1076	1076	Retrospective observational study with ropensity matching	Not specified	N/R	Late stroke, POAF	1.3 years (mean)

Abbreviations: OAC: oral anticoagulation; LAA: left atrial appendage; LAAO: left atrial appendage occlusion; POAF: postoperative atrial fibrillation; ND: not determined; NR: not reported.

### 3.3 Risk of bias assessment

A qualitative assessment was performed using the ROBINS-I and the RoB2 tools. The results are presented in Supplementary Material 2. Baseline characteristics of interest were not clearly reported in the article by Kim et al., making the study susceptible to a high risk of bias due to confounding [16].

### 3.4 Patient characteristics

After excluding non-matched patients, data from a maximum of 7369 patients were available for analysis. Baseline clinical characteristics are reported in Supplementary Material 3. The pooled mean age for the studies exploring the effect of prophylactic LAAO was 64.8 years. The majority of the patients were male: 69.9 % (4779/6835) (data was available for 6835/7369 patients). Eight out of 10 studies included only patients with no history of AF. Nagpal et al. and Park-Hansen et al. did not exclude patients with a history of AF, but in their studies the proportion of patients with a history of AF in both the treatment and control arm did not exceed 20 % and was comparable between groups [20,21].

### 3.5 Intra-operative characteristics

Intra-operative surgical characteristics across the studies are reported in Supplementary Material 3. Three of the 10 studies exploring the effect of prophylactic LAAO included 1341 patients who underwent isolated mitral valve surgery. In the remaining studies, the majority of patients (4316/6028, 71.6 %) underwent coronary artery bypass surgery.

### 3.6 Early outcomes

**Fig. 2:**
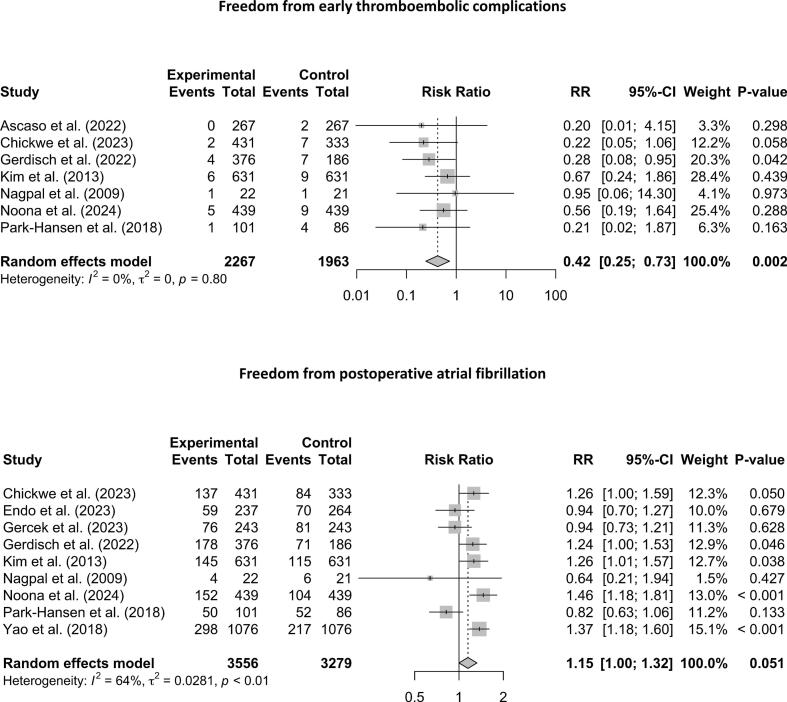
Pooled analysis for early thromboembolic complications (above). The net effect of LAAO was a reduction in early thromboembolic complications. However, LAAO was associated with an increase in POAF (below), albeit statistically not significant.

Seven of the 10 studies on prophylactic LAAO reported data on early thromboembolic complications. The combined incidence of early post-operative thromboembolic events (Fig. 2) was 1.4 % (58/4230). Prophylactic LAAO was associated with a significant risk reduction of 58 % in early thromboembolic events (RR: 0.42; 95 % CI: 0.25 to 0.73; *p* = 0.002; I^2^ = 0 %). On the other hand, the association between LAAO and POAF (Fig. 2) failed to reach statistical significance (RR: 1.15; 95 % CI: 1.00 to 1.32; *p* = 0.051; I^2^ = 64 %).

### 3.7 Secondary outcomes

**Fig. 3:**
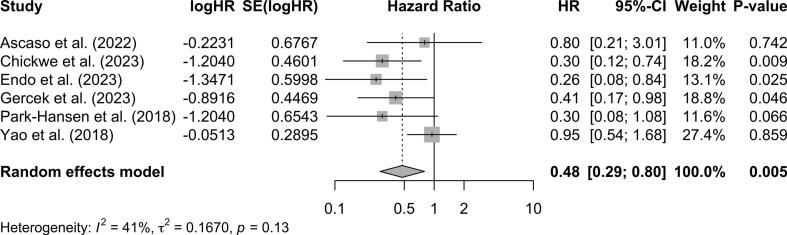
Pooled analysis for all-time thromboembolic complications. LAAO demonstrates a significant effect on the reduction of all-time thromboembolic complications.

**Fig. 4:**
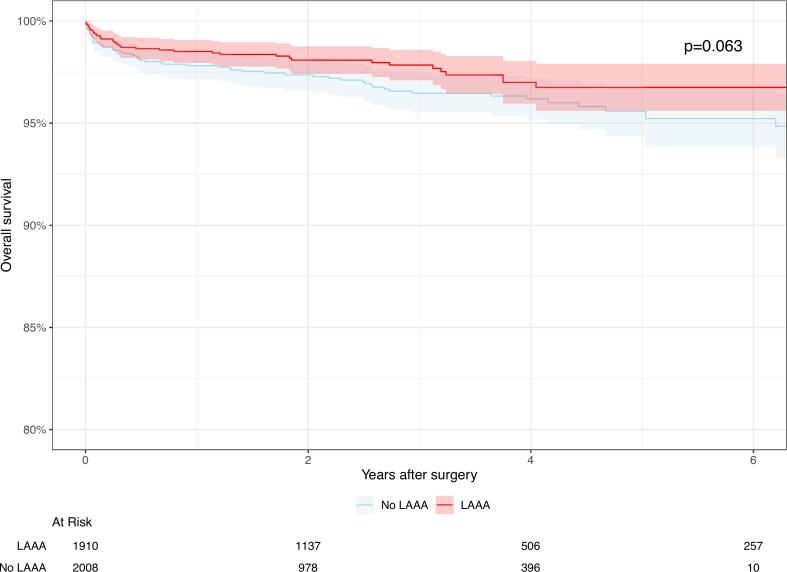
Reconstructed individual patient data Kaplan-Meier survival curves on freedom from thromboembolic complications.

Prophylactic LAAO was also associated with a significant risk reduction of 52 % in all-time thromboembolic complications (HR: 0.48; 95 % CI: 0.29 to 0.80; *p* = 0.005; I^2^ = 41 %) (Fig. 3). The Kaplan-Meier freedom from thromboembolic complication curves using reconstructed IPD are presented in Fig. 4. At 6 years after surgery, the estimated freedom from thromboembolic complications was higher in the LAAO group, 96.7 % (95 % CI 95.6–97.9 %) and 95.2 % (95 % CI 93.8–96.6 %) for the LAAO and no LAAO groups, respectively, but the difference just failed to reach statistical significance (*p* = 0.063). Two of the 10 studies on prophylactic LAAO reported data on late AF. In the article by Chikwe et al., 1.4 % (6/431) in the LAAO group developed late AF in comparison to 4.2 % (14/333) in the control group (*p* = 0.02). Furthermore, Ascaso et al. reported that there was no statistically significant association between late AF and LAAO (HR: 5.02; 95 % CI: 0.58 to 42.81; *p* = 0.14) [10].

## 4 Discussion

The main findings of the present meta-analysis are: 1) in the early period after cardiac surgery, prophylactic LAAO seemed associated with a significant decrease in risk of postoperative thromboembolic events; 2) a correlation between prophylactic concomitant LAAO and POAF might be present but we failed to observe a statistically significant correlation; 3) prophylactic LAAO seemed to reduce the all-time risk of postoperative thromboembolic events.

The beneficial effect of LAAO in patients undergoing cardiac surgery with known AF has recently been proven in the Left Atrial Appendage Occlusion Study (LAAOS) III trial [9,22]. In this trial, LAAO reduced the risk of thromboembolic complications by 18 % early after surgery, whereafter a larger reduction in the late risk of thromboembolic complications, consistent over the years of follow-up, was observed. In light of these positive results, prophylactic LAAO might be appropriate in patients with no known AF as a preventative measure and has already been applied in practice by a number of surgeons [23].

In our results, we observed that LAAO was associated with a 58 % decrease of early thromboembolic events and a 52 % decrease of all-time thromboembolic events. This indicates that following surgery most thromboembolic complications originated from the LAA and suggests that air embolism, intra-operative debris, and aortic cross-clamping were not the main underlying mechanism behind early thromboembolic complications. Although prophylactic LAAO appears to be effective at preventing post-operative thromboembolic events early after cardiac surgery in the studied population, its effects in the long term seems of limited clinical significance as the risk of thromboembolic events declined steeply early after surgery in the studied population. Thus, the observed benefits of prophylactic LAAO in patients with no known AF seem at this point different than in patients with known AF.

Although LAAO appears to prevent early thromboembolic complications, LAAO is not without risks. The possibility of intraoperative complications is low but the possibility of damaging the circumflex artery or postoperative bleeding, depending on the technique used, need to be kept in mind [24,25]. Moreover, POAF tended to occur more often following LAAO, but the difference was not statistically significant. Closure of the LAA includes cardiac mobilization, leads to ischemia of the left atrial tissue and, depending on the technique used, does not always result in electrical isolation of the LAA. Moreover, the LAA is the most compliant part of the left atrium (LA) and forms a volume reserve to prevent acute rises in pressure within the LA [26]. This could lead to a higher susceptibility to POAF in postoperative patients.

Should an increased risk of AF persist late after surgery, this would present an important side effect of prophylactic LAAO. While the study by Chikwe et al. did not observe a higher risk of late AF, Ascaso et al. reported a fivefold increase in late AF [10,11]. The study by Ascaso et al. focused on patients undergoing mitral valve surgery and recurrent mitral regurgitation was seen significantly more often in the LAAO group. This provides an alternative explanation for the higher frequency of late AF in this group [10]. Technically sound LAAO will result not only in volume reduction of the LAA but also electrical isolation and will, theoretically, reduce the number of potential ectopic foci responsible for AF induction [27,28]. Therefore, a positive effect on the reduction of de novo AF could even be expected. Moreover, additional benefits of LAAO, including favorable effect on the neurohormonal and homeostasis systems, could provide additional benefits in the population of patients undergoing cardiac surgery, who are also at risk of developing other cardiovascular diseases [[29], [30], [31]].

Various surgical techniques were used for LAAO in the included studies and the technique used was sometimes not clearly defined. As demonstrated by Kanderian et al., LAAO appears to be more prone to failure when performed by suture closure compared to surgical excision [32]. Unsuccessful LAA closure and LAA recanalization are particularly concerning because they increase the risk of thrombogenesis [33]. This is, however, demonstrated on patients with known AF, also suffering from endothelial dysfunction and left atrial dilation with intra-atrial blood flow alterations, and might not be of clinical significance in patient in sinus rhythm, in whom the risk of late thromboembolic complications is low, as demonstrated.

Recently, there has been a significant rise in the interest in prophylactic LAAO and pulmonary vein isolation during cardiac surgery in patients with no known AF [36,37]. This could provide a valuable addition to standard cardiac surgical care in the future, enhancing the safety profile of the procedure and potentially enabling earlier hospital discharge.

### 4.1 Study limitations

The present study is subject to inherent limitations of the included RCTs and retrospective observational studies. Since most of the studies were retrospective and not all employed propensity score matching or other comparable statistical methods to account for the lack of patient randomization, the results of our analysis may be subject to bias. Different types of LAAO techniques were performed throughout the studies but were inevitably assessed as one entity. Importantly, no studies have systematically assessed the success rate of LAAO or reported the incidence of incomplete occlusion, despite the fact that several techniques used may predispose to LAA recanalization. Moreover, the complication rate, including myocardial infarction and postoperative bleeding associated with the intervention, was poorly reported. Lastly, the association between LAAO and POAF failed to reach statistical significance, a finding that might be related to the number of patients and events.

## 5 Conclusions

Prophylactic LAAO seemed associated with a lower risk of early postoperative thromboembolic complications. However, an association between LAAO and POAF might be present, but our results failed to demonstrate a significant association. In patients undergoing cardiac surgery with no known history of AF, late thromboembolic complications were uncommon.

## CRediT authorship contribution statement

**Chengji Zhao:** Writing – original draft, Methodology, Data curation, Conceptualization. **Evaldas Girdauskas:** Writing – review & editing, Supervision. **Jan W. Schoones:** Writing – review & editing, Methodology, Data curation. **Robert J.M. Klautz:** Writing – review & editing, Supervision, Resources. **Meindert Palmen:** Writing – original draft, Supervision, Methodology, Conceptualization. **Anton Tomšič:** Writing – original draft, Methodology, Formal analysis, Data curation, Conceptualization.

## Ethical committee approval

No approval of the ethical committee was needed due to the nature of this study (systematic review and meta-analysis).

## Funding

No funding.

## Declaration of competing interest

None.

## Data Availability

All data used in the current meta-analysis have been extracted from previously published articles.

## Appendix A Supplementary data

Supplementary material Image 1
