# Supplementary material for: The clinical impact of prophylactic concomitant left atrial appendage occlusion during cardiac surgery: A systematic review and meta-analysis
Source: Am Heart J Plus. 2025 Mar 26;53:100534. doi: 10.1016/j.ahjo.2025.100534 (PMC11987691; doi:10.1016/j.ahjo.2025.100534)
Supplement: Supplementary file 1 — Supplementary material [file mmc1.docx]

**Supplementary Material 1: Full search**

**PubMed**

https://pubmed.ncbi.nlm.nih.gov/

**(**(("Stroke"[Mesh] OR "Ischemic Attack, Transient"[Mesh] OR "Stroke"[tw] OR "Apoplexy"[tw] OR "Brain Vascular Accident"[tw] OR "Brain Vascular Accidents"[tw] OR "Cerebrovascular Accident"[tw] OR "Cerebrovascular Accident"[tw] OR "Cerebrovascular Accidents"[tw] OR "Cerebrovascular Accidents"[tw] OR "CVA"[tw] OR "CVAs"[tw] OR "Brain Infarction"[tw] OR "Brain Infarctions"[tw] OR "Brain Stem Infarction"[tw] OR "Brain Stem Infarctions"[tw] OR "CADASIL"[tw] OR "Cerebral Artery Infarction"[tw] OR "Cerebral Artery Infarctions"[tw] OR "Cerebral Infarction"[tw] OR "Cerebral Infarctions"[tw] OR "Lateral Medullary Syndrome"[tw] OR "Multi-Infarct Dementia"[tw] OR "transient ischemic attack"[tw] OR "TIA"[tw] OR "TIAs"[tw] OR "Transient Brainstem Ischemia"[tw] OR "Transient Cerebral Ischemia"[tw] OR "Transient Cerebral Ischemias"[tw] OR "Transient Ischemic Attacks"[tw] OR "Transient Brainstem Ischaemia"[tw] OR "Transient Cerebral Ischaemia"[tw] OR "Transient Ischaemic Attack"[tw] OR "Transient Ischaemic Attacks"[tw] OR "Brain Vascular Accident"[title/abstract:~3] OR "Brain Vascular Accidents"[title/abstract:~3] OR "Cerebrovascular Accident"[title/abstract:~3] OR "Cerebrovascular Accident"[title/abstract:~3] OR "Cerebrovascular Accidents"[title/abstract:~3] OR "Cerebrovascular Accidents"[title/abstract:~3] OR "Brain Infarction"[title/abstract:~3] OR "Brain Infarctions"[title/abstract:~3] OR "Brain Stem Infarction"[title/abstract:~3] OR "Brain Stem Infarctions"[title/abstract:~3] OR "Cerebral Artery Infarction"[title/abstract:~3] OR "Cerebral Artery Infarctions"[title/abstract:~3] OR "Cerebral Infarction"[title/abstract:~3] OR "Cerebral Infarctions"[title/abstract:~3] OR "Lateral Medullary Syndrome"[title/abstract:~3] OR "Multi-Infarct Dementia"[title/abstract:~3] OR "transient ischemic attack"[title/abstract:~3] OR "Transient Brainstem Ischemia"[title/abstract:~3] OR "Transient Brainstem Ischemias"[title/abstract:~3] OR "Transient Cerebral Ischemia"[title/abstract:~3] OR "Transient Cerebral Ischemias"[title/abstract:~3] OR "Transient Ischemic Attacks"[title/abstract:~3] OR "Transient Brainstem Ischaemias"[title/abstract:~3] OR "Transient Brainstem Ischaemia"[title/abstract:~3] OR "Transient Cerebral Ischaemia"[title/abstract:~3] OR "Transient Cerebral Ischaemias"[title/abstract:~3] OR "Transient Ischaemic Attack"[title/abstract:~3] OR "Transient Ischaemic Attacks"[title/abstract:~3]) AND ("Atrial Appendage"[Mesh] OR "Atrial Appendage"[tw] OR "Atrial Appendages"[tw] OR "Atrium Appendage"[tw] OR "Atrium Appendages"[tw] OR "Ablation Techniques"[Mesh] OR "Left Atrial Appendage*"[tw] OR "LAA"[tw] OR "LAAs"[tw] OR "ablation"[tw] OR "ablat*"[tw] OR "pulmonary vein isolation"[tw] OR "PVI"[tw] OR "Atrial Appendage"[title/abstract:~3] OR "Atrial Appendages"[title/abstract:~3] OR "Atrium Appendage"[title/abstract:~3] OR "Atrium Appendages"[title/abstract:~3] OR "pulmonary vein isolation"[title/abstract:~3]) AND ("Mitral Valve Annuloplasty"[Mesh] OR "Mitral Valve Prolapse"[Mesh] OR "Mitral Valve Annuloplasty"[tw] OR "Mitral Valve Prolapse"[tw] OR "Mitral Annuloplasty"[tw] OR "Mitral Prolapse"[tw] OR "Mitral valve"[tw] OR "Mitral valves"[tw] OR "Mitral valve*"[tw] OR "MVP"[tw] OR "MVr"[tw] OR "Mitral valve"[title/abstract:~3] OR "Mitral valves"[title/abstract:~3] OR "Mitral Annuloplasty"[title/abstract:~3] OR "Mitral Prolapse"[title/abstract:~3] **OR (("Cardiac Surgical Procedures"[majr:noexp] OR "Cardiac Surgery"[ti]) AND ("valve"[tw] OR "valves"[tw]))**)) **OR** (("prophylactic"[tw] OR "prophyla*"[tw]) AND ("Atrial Appendage"[Mesh] OR "Atrial Appendage"[tw] OR "Atrial Appendages"[tw] OR "Atrium Appendage"[tw] OR "Atrium Appendages"[tw] OR "Ablation Techniques"[Mesh] OR "Left Atrial Appendage*"[tw] OR "LAA"[tw] OR "LAAs"[tw] OR "ablation"[tw] OR "ablat*"[tw] OR "pulmonary vein isolation"[tw] OR "PVI"[tw] OR "Atrial Appendage"[title/abstract:~3] OR "Atrial Appendages"[title/abstract:~3] OR "Atrium Appendage"[title/abstract:~3] OR "Atrium Appendages"[title/abstract:~3] OR "pulmonary vein isolation"[title/abstract:~3]) AND ("Mitral Valve Annuloplasty"[Mesh] OR "Mitral Valve Prolapse"[Mesh] OR "Mitral Valve Annuloplasty"[tw] OR "Mitral Valve Prolapse"[tw] OR "Mitral Annuloplasty"[tw] OR "Mitral Prolapse"[tw] OR "Mitral valve"[tw] OR "Mitral valves"[tw] OR "Mitral valve*"[tw] OR "MVP"[tw] OR "MVr"[tw] OR "Mitral valve"[title/abstract:~3] OR "Mitral valves"[title/abstract:~3] OR "Mitral Annuloplasty"[title/abstract:~3] OR "Mitral Prolapse"[title/abstract:~3] **OR "Cardiac Surgical Procedures"[majr:noexp] OR "Cardiac Surgery"[ti] OR "coronary artery bypass grafting"[ti] OR "Coronary Artery Bypass"[majr] OR "CABG"[ti]**))**)** NOT (("Case Reports"[ptyp] OR "case report"[ti] OR "case rep"[all fields] OR "Review"[ptyp] OR "review"[ti]) NOT ("Clinical Study"[ptyp] OR "trial"[ti] OR "RCT"[ti]))

**Embase**

<http://ovidsp.ovid.com/ovidweb.cgi?T=JS&PAGE=main&MODE=ovid&D=oemezd>

**(**((exp *"Cerebrovascular Accident"/ OR exp *"Transient Ischemic Attack"/ OR "Stroke".ti,ab OR "Apoplexy".ti,ab OR "Brain Vascular Accident".ti,ab OR "Brain Vascular Accidents".ti,ab OR "Cerebrovascular Accident".ti,ab OR "Cerebrovascular Accident".ti,ab OR "Cerebrovascular Accidents".ti,ab OR "Cerebrovascular Accidents".ti,ab OR "CVA".ti,ab OR "CVAs".ti,ab OR "Brain Infarction".ti,ab OR "Brain Infarctions".ti,ab OR "Brain Stem Infarction".ti,ab OR "Brain Stem Infarctions".ti,ab OR "CADASIL".ti,ab OR "Cerebral Artery Infarction".ti,ab OR "Cerebral Artery Infarctions".ti,ab OR "Cerebral Infarction".ti,ab OR "Cerebral Infarctions".ti,ab OR "Lateral Medullary Syndrome".ti,ab OR "Multi-Infarct Dementia".ti,ab OR "transient ischemic attack".ti,ab OR "TIA".ti,ab OR "TIAs".ti,ab OR "Transient Brainstem Ischemia".ti,ab OR "Transient Cerebral Ischemia".ti,ab OR "Transient Cerebral Ischemias".ti,ab OR "Transient Ischemic Attacks".ti,ab OR "Transient Brainstem Ischaemia".ti,ab OR "Transient Cerebral Ischaemia".ti,ab OR "Transient Ischaemic Attack".ti,ab OR "Transient Ischaemic Attacks".ti,ab OR ("Brain" ADJ3 "Vascular" ADJ3 "Accident").ti,ab OR ("Brain" ADJ3 "Vascular" ADJ3 "Accidents").ti,ab OR ("Cerebrovascular" ADJ3 "Accident").ti,ab OR ("Cerebrovascular" ADJ3 "Accident").ti,ab OR ("Cerebrovascular" ADJ3 "Accidents").ti,ab OR ("Cerebrovascular" ADJ3 "Accidents").ti,ab OR ("Brain" ADJ3 "Infarction").ti,ab OR ("Brain" ADJ3 "Infarctions").ti,ab OR ("Brain" ADJ3 "Stem" ADJ3 "Infarction").ti,ab OR ("Brain" ADJ3 "Stem" ADJ3 "Infarctions").ti,ab OR ("Cerebral" ADJ3 "Artery" ADJ3 "Infarction").ti,ab OR ("Cerebral" ADJ3 "Artery" ADJ3 "Infarctions").ti,ab OR ("Cerebral" ADJ3 "Infarction").ti,ab OR ("Cerebral" ADJ3 "Infarctions").ti,ab OR ("Lateral" ADJ3 "Medullary" ADJ3 "Syndrome").ti,ab OR ("Multi-Infarct" ADJ3 "Dementia").ti,ab OR ("transient" ADJ3 "ischemic" ADJ3 "attack").ti,ab OR ("Transient" ADJ3 "Brainstem" ADJ3 "Ischemia").ti,ab OR ("Transient" ADJ3 "Brainstem" ADJ3 "Ischemias").ti,ab OR ("Transient" ADJ3 "Cerebral" ADJ3 "Ischemia").ti,ab OR ("Transient" ADJ3 "Cerebral" ADJ3 "Ischemias").ti,ab OR ("Transient" ADJ3 "Ischemic" ADJ3 "Attacks").ti,ab OR ("Transient" ADJ3 "Brainstem" ADJ3 "Ischaemias").ti,ab OR ("Transient" ADJ3 "Brainstem" ADJ3 "Ischaemia").ti,ab OR ("Transient" ADJ3 "Cerebral" ADJ3 "Ischaemia").ti,ab OR ("Transient" ADJ3 "Cerebral" ADJ3 "Ischaemias").ti,ab OR ("Transient" ADJ3 "Ischaemic" ADJ3 "Attack").ti,ab OR ("Transient" ADJ3 "Ischaemic" ADJ3 "Attacks").ti,ab) AND (exp *"Heart Atrium Appendage"/ OR "Atrial Appendage".ti,ab OR "Atrial Appendages".ti,ab OR "Atrium Appendage".ti,ab OR "Atrium Appendages".ti,ab OR exp *"Ablation Therapy"/ OR "Left Atrial Appendage*".ti,ab OR "LAA".ti,ab OR "LAAs".ti,ab OR "ablation".ti,ab OR "ablat*".ti,ab OR "pulmonary vein isolation".ti,ab OR "PVI".ti,ab OR (("Atrial" ADJ3 "Appendage") OR ("Atrial" ADJ3 "Appendages") OR ("Atrium" ADJ3 "Appendage") OR ("Atrium" ADJ3 "Appendages") OR ("pulmonary" ADJ3 "vein" ADJ3 "isolation")).ti,ab) AND (exp *"Mitral Annuloplasty"/ OR exp *"Mitral Valve Prolapse"/ OR "Mitral Valve Annuloplasty".ti,ab OR "Mitral Valve Prolapse".ti,ab OR "Mitral Annuloplasty".ti,ab OR "Mitral Prolapse".ti,ab OR "Mitral valve".ti,ab OR "Mitral valves".ti,ab OR "Mitral valve*".ti,ab OR "MVP".ti,ab OR "MVr".ti,ab OR (("Mitral" ADJ3 "valve") OR ("Mitral" ADJ3 "valves") OR ("Mitral" ADJ3 "Annuloplasty") OR ("Mitral" ADJ3 "Prolapse")).ti,ab **OR ((*"Heart Surgery"/ OR "Cardiac Surgery".ti) AND ("valve".ti,ab OR "valves".ti,ab))**)) **OR** (("prophylactic".ti,ab OR "prophyla*".ti,ab) AND (exp *"Heart Atrium Appendage"/ OR "Atrial Appendage".ti,ab OR "Atrial Appendages".ti,ab OR "Atrium Appendage".ti,ab OR "Atrium Appendages".ti,ab OR exp *"Ablation Therapy"/ OR "Left Atrial Appendage*".ti,ab OR "LAA".ti,ab OR "LAAs".ti,ab OR "ablation".ti,ab OR "ablat*".ti,ab OR "pulmonary vein isolation".ti,ab OR "PVI".ti,ab OR (("Atrial" ADJ3 "Appendage") OR ("Atrial" ADJ3 "Appendages") OR ("Atrium" ADJ3 "Appendage") OR ("Atrium" ADJ3 "Appendages") OR ("pulmonary" ADJ3 "vein" ADJ3 "isolation")).ti,ab) AND (exp *"Mitral Annuloplasty"/ OR exp *"Mitral Valve Prolapse"/ OR "Mitral Valve Annuloplasty".ti,ab OR "Mitral Valve Prolapse".ti,ab OR "Mitral Annuloplasty".ti,ab OR "Mitral Prolapse".ti,ab OR "Mitral valve".ti,ab OR "Mitral valves".ti,ab OR "Mitral valve*".ti,ab OR "MVP".ti,ab OR "MVr".ti,ab OR (("Mitral" ADJ3 "valve") OR ("Mitral" ADJ3 "valves") OR ("Mitral" ADJ3 "Annuloplasty") OR ("Mitral" ADJ3 "Prolapse")).ti,ab **OR *"Cardiac Surgery"/ OR "Cardiac Surgery".ti**))**)** NOT (("Case Report"/ OR "case report".ti OR ("case" AND ("report" OR "reports")).jx OR exp "Review"/ OR "review".ti) NOT ("Clinical Study"/ OR exp "Clinical Trial"/ OR "trial".ti OR "RCT".ti)) NOT (conference review or conference abstract).pt

**Web of Science**

<http://isiknowledge.com/wos>

**(**((TI=("Cerebrovascular Accident" OR "Transient Ischemic Attack" OR "Stroke" OR "Apoplexy" OR "Brain Vascular Accident" OR "Brain Vascular Accidents" OR "Cerebrovascular Accident" OR "Cerebrovascular Accident" OR "Cerebrovascular Accidents" OR "Cerebrovascular Accidents" OR "CVA" OR "CVAs" OR "Brain Infarction" OR "Brain Infarctions" OR "Brain Stem Infarction" OR "Brain Stem Infarctions" OR "CADASIL" OR "Cerebral Artery Infarction" OR "Cerebral Artery Infarctions" OR "Cerebral Infarction" OR "Cerebral Infarctions" OR "Lateral Medullary Syndrome" OR "Multi-Infarct Dementia" OR "transient ischemic attack" OR "TIA" OR "TIAs" OR "Transient Brainstem Ischemia" OR "Transient Cerebral Ischemia" OR "Transient Cerebral Ischemias" OR "Transient Ischemic Attacks" OR "Transient Brainstem Ischaemia" OR "Transient Cerebral Ischaemia" OR "Transient Ischaemic Attack" OR "Transient Ischaemic Attacks" OR ("Brain" NEAR/3 "Vascular" NEAR/3 "Accident") OR ("Brain" NEAR/3 "Vascular" NEAR/3 "Accidents") OR ("Cerebrovascular" NEAR/3 "Accident") OR ("Cerebrovascular" NEAR/3 "Accident") OR ("Cerebrovascular" NEAR/3 "Accidents") OR ("Cerebrovascular" NEAR/3 "Accidents") OR ("Brain" NEAR/3 "Infarction") OR ("Brain" NEAR/3 "Infarctions") OR ("Brain" NEAR/3 "Stem" NEAR/3 "Infarction") OR ("Brain" NEAR/3 "Stem" NEAR/3 "Infarctions") OR ("Cerebral" NEAR/3 "Artery" NEAR/3 "Infarction") OR ("Cerebral" NEAR/3 "Artery" NEAR/3 "Infarctions") OR ("Cerebral" NEAR/3 "Infarction") OR ("Cerebral" NEAR/3 "Infarctions") OR ("Lateral" NEAR/3 "Medullary" NEAR/3 "Syndrome") OR ("Multi-Infarct" NEAR/3 "Dementia") OR ("transient" NEAR/3 "ischemic" NEAR/3 "attack") OR ("Transient" NEAR/3 "Brainstem" NEAR/3 "Ischemia") OR ("Transient" NEAR/3 "Brainstem" NEAR/3 "Ischemias") OR ("Transient" NEAR/3 "Cerebral" NEAR/3 "Ischemia") OR ("Transient" NEAR/3 "Cerebral" NEAR/3 "Ischemias") OR ("Transient" NEAR/3 "Ischemic" NEAR/3 "Attacks") OR ("Transient" NEAR/3 "Brainstem" NEAR/3 "Ischaemias") OR ("Transient" NEAR/3 "Brainstem" NEAR/3 "Ischaemia") OR ("Transient" NEAR/3 "Cerebral" NEAR/3 "Ischaemia") OR ("Transient" NEAR/3 "Cerebral" NEAR/3 "Ischaemias") OR ("Transient" NEAR/3 "Ischaemic" NEAR/3 "Attack") OR ("Transient" NEAR/3 "Ischaemic" NEAR/3 "Attacks")) OR AK=("Cerebrovascular Accident" OR "Transient Ischemic Attack" OR "Stroke" OR "Apoplexy" OR "Brain Vascular Accident" OR "Brain Vascular Accidents" OR "Cerebrovascular Accident" OR "Cerebrovascular Accident" OR "Cerebrovascular Accidents" OR "Cerebrovascular Accidents" OR "CVA" OR "CVAs" OR "Brain Infarction" OR "Brain Infarctions" OR "Brain Stem Infarction" OR "Brain Stem Infarctions" OR "CADASIL" OR "Cerebral Artery Infarction" OR "Cerebral Artery Infarctions" OR "Cerebral Infarction" OR "Cerebral Infarctions" OR "Lateral Medullary Syndrome" OR "Multi-Infarct Dementia" OR "transient ischemic attack" OR "TIA" OR "TIAs" OR "Transient Brainstem Ischemia" OR "Transient Cerebral Ischemia" OR "Transient Cerebral Ischemias" OR "Transient Ischemic Attacks" OR "Transient Brainstem Ischaemia" OR "Transient Cerebral Ischaemia" OR "Transient Ischaemic Attack" OR "Transient Ischaemic Attacks" OR ("Brain" NEAR/3 "Vascular" NEAR/3 "Accident") OR ("Brain" NEAR/3 "Vascular" NEAR/3 "Accidents") OR ("Cerebrovascular" NEAR/3 "Accident") OR ("Cerebrovascular" NEAR/3 "Accident") OR ("Cerebrovascular" NEAR/3 "Accidents") OR ("Cerebrovascular" NEAR/3 "Accidents") OR ("Brain" NEAR/3 "Infarction") OR ("Brain" NEAR/3 "Infarctions") OR ("Brain" NEAR/3 "Stem" NEAR/3 "Infarction") OR ("Brain" NEAR/3 "Stem" NEAR/3 "Infarctions") OR ("Cerebral" NEAR/3 "Artery" NEAR/3 "Infarction") OR ("Cerebral" NEAR/3 "Artery" NEAR/3 "Infarctions") OR ("Cerebral" NEAR/3 "Infarction") OR ("Cerebral" NEAR/3 "Infarctions") OR ("Lateral" NEAR/3 "Medullary" NEAR/3 "Syndrome") OR ("Multi-Infarct" NEAR/3 "Dementia") OR ("transient" NEAR/3 "ischemic" NEAR/3 "attack") OR ("Transient" NEAR/3 "Brainstem" NEAR/3 "Ischemia") OR ("Transient" NEAR/3 "Brainstem" NEAR/3 "Ischemias") OR ("Transient" NEAR/3 "Cerebral" NEAR/3 "Ischemia") OR ("Transient" NEAR/3 "Cerebral" NEAR/3 "Ischemias") OR ("Transient" NEAR/3 "Ischemic" NEAR/3 "Attacks") OR ("Transient" NEAR/3 "Brainstem" NEAR/3 "Ischaemias") OR ("Transient" NEAR/3 "Brainstem" NEAR/3 "Ischaemia") OR ("Transient" NEAR/3 "Cerebral" NEAR/3 "Ischaemia") OR ("Transient" NEAR/3 "Cerebral" NEAR/3 "Ischaemias") OR ("Transient" NEAR/3 "Ischaemic" NEAR/3 "Attack") OR ("Transient" NEAR/3 "Ischaemic" NEAR/3 "Attacks")) OR AB=("Cerebrovascular Accident" OR "Transient Ischemic Attack" OR "Stroke" OR "Apoplexy" OR "Brain Vascular Accident" OR "Brain Vascular Accidents" OR "Cerebrovascular Accident" OR "Cerebrovascular Accident" OR "Cerebrovascular Accidents" OR "Cerebrovascular Accidents" OR "CVA" OR "CVAs" OR "Brain Infarction" OR "Brain Infarctions" OR "Brain Stem Infarction" OR "Brain Stem Infarctions" OR "CADASIL" OR "Cerebral Artery Infarction" OR "Cerebral Artery Infarctions" OR "Cerebral Infarction" OR "Cerebral Infarctions" OR "Lateral Medullary Syndrome" OR "Multi-Infarct Dementia" OR "transient ischemic attack" OR "TIA" OR "TIAs" OR "Transient Brainstem Ischemia" OR "Transient Cerebral Ischemia" OR "Transient Cerebral Ischemias" OR "Transient Ischemic Attacks" OR "Transient Brainstem Ischaemia" OR "Transient Cerebral Ischaemia" OR "Transient Ischaemic Attack" OR "Transient Ischaemic Attacks" OR ("Brain" NEAR/3 "Vascular" NEAR/3 "Accident") OR ("Brain" NEAR/3 "Vascular" NEAR/3 "Accidents") OR ("Cerebrovascular" NEAR/3 "Accident") OR ("Cerebrovascular" NEAR/3 "Accident") OR ("Cerebrovascular" NEAR/3 "Accidents") OR ("Cerebrovascular" NEAR/3 "Accidents") OR ("Brain" NEAR/3 "Infarction") OR ("Brain" NEAR/3 "Infarctions") OR ("Brain" NEAR/3 "Stem" NEAR/3 "Infarction") OR ("Brain" NEAR/3 "Stem" NEAR/3 "Infarctions") OR ("Cerebral" NEAR/3 "Artery" NEAR/3 "Infarction") OR ("Cerebral" NEAR/3 "Artery" NEAR/3 "Infarctions") OR ("Cerebral" NEAR/3 "Infarction") OR ("Cerebral" NEAR/3 "Infarctions") OR ("Lateral" NEAR/3 "Medullary" NEAR/3 "Syndrome") OR ("Multi-Infarct" NEAR/3 "Dementia") OR ("transient" NEAR/3 "ischemic" NEAR/3 "attack") OR ("Transient" NEAR/3 "Brainstem" NEAR/3 "Ischemia") OR ("Transient" NEAR/3 "Brainstem" NEAR/3 "Ischemias") OR ("Transient" NEAR/3 "Cerebral" NEAR/3 "Ischemia") OR ("Transient" NEAR/3 "Cerebral" NEAR/3 "Ischemias") OR ("Transient" NEAR/3 "Ischemic" NEAR/3 "Attacks") OR ("Transient" NEAR/3 "Brainstem" NEAR/3 "Ischaemias") OR ("Transient" NEAR/3 "Brainstem" NEAR/3 "Ischaemia") OR ("Transient" NEAR/3 "Cerebral" NEAR/3 "Ischaemia") OR ("Transient" NEAR/3 "Cerebral" NEAR/3 "Ischaemias") OR ("Transient" NEAR/3 "Ischaemic" NEAR/3 "Attack") OR ("Transient" NEAR/3 "Ischaemic" NEAR/3 "Attacks"))) AND (TI=("Heart Atrium Appendage" OR "Atrial Appendage" OR "Atrial Appendages" OR "Atrium Appendage" OR "Atrium Appendages" OR "Ablation Therapy" OR "Left Atrial Appendage*" OR "LAA" OR "LAAs" OR "ablation" OR "ablat*" OR "pulmonary vein isolation" OR "PVI" OR (("Atrial" NEAR/3 "Appendage") OR ("Atrial" NEAR/3 "Appendages") OR ("Atrium" NEAR/3 "Appendage") OR ("Atrium" NEAR/3 "Appendages") OR ("pulmonary" NEAR/3 "vein" NEAR/3 "isolation"))) OR AK=("Heart Atrium Appendage" OR "Atrial Appendage" OR "Atrial Appendages" OR "Atrium Appendage" OR "Atrium Appendages" OR "Ablation Therapy" OR "Left Atrial Appendage*" OR "LAA" OR "LAAs" OR "ablation" OR "ablat*" OR "pulmonary vein isolation" OR "PVI" OR (("Atrial" NEAR/3 "Appendage") OR ("Atrial" NEAR/3 "Appendages") OR ("Atrium" NEAR/3 "Appendage") OR ("Atrium" NEAR/3 "Appendages") OR ("pulmonary" NEAR/3 "vein" NEAR/3 "isolation"))) OR AB=("Heart Atrium Appendage" OR "Atrial Appendage" OR "Atrial Appendages" OR "Atrium Appendage" OR "Atrium Appendages" OR "Ablation Therapy" OR "Left Atrial Appendage*" OR "LAA" OR "LAAs" OR "ablation" OR "ablat*" OR "pulmonary vein isolation" OR "PVI" OR (("Atrial" NEAR/3 "Appendage") OR ("Atrial" NEAR/3 "Appendages") OR ("Atrium" NEAR/3 "Appendage") OR ("Atrium" NEAR/3 "Appendages") OR ("pulmonary" NEAR/3 "vein" NEAR/3 "isolation")))) AND (TI=("Mitral Annuloplasty" OR "Mitral Valve Prolapse" OR "Mitral Valve Annuloplasty" OR "Mitral Valve Prolapse" OR "Mitral Annuloplasty" OR "Mitral Prolapse" OR "Mitral valve" OR "Mitral valves" OR "Mitral valve*" OR "MVP" OR "MVr" OR (("Mitral" NEAR/3 "valve") OR ("Mitral" NEAR/3 "valves") OR ("Mitral" NEAR/3 "Annuloplasty") OR ("Mitral" NEAR/3 "Prolapse"))) OR AK=("Mitral Annuloplasty" OR "Mitral Valve Prolapse" OR "Mitral Valve Annuloplasty" OR "Mitral Valve Prolapse" OR "Mitral Annuloplasty" OR "Mitral Prolapse" OR "Mitral valve" OR "Mitral valves" OR "Mitral valve*" OR "MVP" OR "MVr" OR (("Mitral" NEAR/3 "valve") OR ("Mitral" NEAR/3 "valves") OR ("Mitral" NEAR/3 "Annuloplasty") OR ("Mitral" NEAR/3 "Prolapse"))) OR AB=("Mitral Annuloplasty" OR "Mitral Valve Prolapse" OR "Mitral Valve Annuloplasty" OR "Mitral Valve Prolapse" OR "Mitral Annuloplasty" OR "Mitral Prolapse" OR "Mitral valve" OR "Mitral valves" OR "Mitral valve*" OR "MVP" OR "MVr" OR (("Mitral" NEAR/3 "valve") OR ("Mitral" NEAR/3 "valves") OR ("Mitral" NEAR/3 "Annuloplasty") OR ("Mitral" NEAR/3 "Prolapse"))) **OR (TI=("Heart Surgery" OR "Cardiac Surgery") AND TS=("valve" OR "valves"))**)) **OR** (TS=("prophylactic" OR "prophyla*") AND (TI=("Heart Atrium Appendage" OR "Atrial Appendage" OR "Atrial Appendages" OR "Atrium Appendage" OR "Atrium Appendages" OR "Ablation Therapy" OR "Left Atrial Appendage*" OR "LAA" OR "LAAs" OR "ablation" OR "ablat*" OR "pulmonary vein isolation" OR "PVI" OR (("Atrial" NEAR/3 "Appendage") OR ("Atrial" NEAR/3 "Appendages") OR ("Atrium" NEAR/3 "Appendage") OR ("Atrium" NEAR/3 "Appendages") OR ("pulmonary" NEAR/3 "vein" NEAR/3 "isolation"))) OR AK=("Heart Atrium Appendage" OR "Atrial Appendage" OR "Atrial Appendages" OR "Atrium Appendage" OR "Atrium Appendages" OR "Ablation Therapy" OR "Left Atrial Appendage*" OR "LAA" OR "LAAs" OR "ablation" OR "ablat*" OR "pulmonary vein isolation" OR "PVI" OR (("Atrial" NEAR/3 "Appendage") OR ("Atrial" NEAR/3 "Appendages") OR ("Atrium" NEAR/3 "Appendage") OR ("Atrium" NEAR/3 "Appendages") OR ("pulmonary" NEAR/3 "vein" NEAR/3 "isolation"))) OR AB=("Heart Atrium Appendage" OR "Atrial Appendage" OR "Atrial Appendages" OR "Atrium Appendage" OR "Atrium Appendages" OR "Ablation Therapy" OR "Left Atrial Appendage*" OR "LAA" OR "LAAs" OR "ablation" OR "ablat*" OR "pulmonary vein isolation" OR "PVI" OR (("Atrial" NEAR/3 "Appendage") OR ("Atrial" NEAR/3 "Appendages") OR ("Atrium" NEAR/3 "Appendage") OR ("Atrium" NEAR/3 "Appendages") OR ("pulmonary" NEAR/3 "vein" NEAR/3 "isolation")))) AND (TS=("Mitral Annuloplasty" OR "Mitral Valve Prolapse" OR "Mitral Valve Annuloplasty" OR "Mitral Valve Prolapse" OR "Mitral Annuloplasty" OR "Mitral Prolapse" OR "Mitral valve" OR "Mitral valves" OR "Mitral valve*" OR "MVP" OR "MVr" OR (("Mitral" NEAR/3 "valve") OR ("Mitral" NEAR/3 "valves") OR ("Mitral" NEAR/3 "Annuloplasty") OR ("Mitral" NEAR/3 "Prolapse"))) OR AK=("Mitral Annuloplasty" OR "Mitral Valve Prolapse" OR "Mitral Valve Annuloplasty" OR "Mitral Valve Prolapse" OR "Mitral Annuloplasty" OR "Mitral Prolapse" OR "Mitral valve" OR "Mitral valves" OR "Mitral valve*" OR "MVP" OR "MVr" OR (("Mitral" NEAR/3 "valve") OR ("Mitral" NEAR/3 "valves") OR ("Mitral" NEAR/3 "Annuloplasty") OR ("Mitral" NEAR/3 "Prolapse"))) OR AB=("Mitral Annuloplasty" OR "Mitral Valve Prolapse" OR "Mitral Valve Annuloplasty" OR "Mitral Valve Prolapse" OR "Mitral Annuloplasty" OR "Mitral Prolapse" OR "Mitral valve" OR "Mitral valves" OR "Mitral valve*" OR "MVP" OR "MVr" OR (("Mitral" NEAR/3 "valve") OR ("Mitral" NEAR/3 "valves") OR ("Mitral" NEAR/3 "Annuloplasty") OR ("Mitral" NEAR/3 "Prolapse"))) **OR** TI=**("Cardiac Surgery" OR "Cardiac Surgery")**))**)** NOT (TI=("Case Report" OR "case" OR "Review") OR AK=("Case Report" OR "Review") OR AB=("case report")) NOT DT=(meeting abstract)

**Cochrane**

<https://www.cochranelibrary.com/advanced-search/search-manager>

**(**(("Cerebrovascular Accident" OR "Transient Ischemic Attack" OR "Stroke" OR "Apoplexy" OR "Brain Vascular Accident" OR "Brain Vascular Accidents" OR "Cerebrovascular Accident" OR "Cerebrovascular Accident" OR "Cerebrovascular Accidents" OR "Cerebrovascular Accidents" OR "CVA" OR "CVAs" OR "Brain Infarction" OR "Brain Infarctions" OR "Brain Stem Infarction" OR "Brain Stem Infarctions" OR "CADASIL" OR "Cerebral Artery Infarction" OR "Cerebral Artery Infarctions" OR "Cerebral Infarction" OR "Cerebral Infarctions" OR "Lateral Medullary Syndrome" OR "Multi-Infarct Dementia" OR "transient ischemic attack" OR "TIA" OR "TIAs" OR "Transient Brainstem Ischemia" OR "Transient Cerebral Ischemia" OR "Transient Cerebral Ischemias" OR "Transient Ischemic Attacks" OR "Transient Brainstem Ischaemia" OR "Transient Cerebral Ischaemia" OR "Transient Ischaemic Attack" OR "Transient Ischaemic Attacks" OR ("Brain" NEAR/3 "Vascular" NEAR/3 "Accident") OR ("Brain" NEAR/3 "Vascular" NEAR/3 "Accidents") OR ("Cerebrovascular" NEAR/3 "Accident") OR ("Cerebrovascular" NEAR/3 "Accident") OR ("Cerebrovascular" NEAR/3 "Accidents") OR ("Cerebrovascular" NEAR/3 "Accidents") OR ("Brain" NEAR/3 "Infarction") OR ("Brain" NEAR/3 "Infarctions") OR ("Brain" NEAR/3 "Stem" NEAR/3 "Infarction") OR ("Brain" NEAR/3 "Stem" NEAR/3 "Infarctions") OR ("Cerebral" NEAR/3 "Artery" NEAR/3 "Infarction") OR ("Cerebral" NEAR/3 "Artery" NEAR/3 "Infarctions") OR ("Cerebral" NEAR/3 "Infarction") OR ("Cerebral" NEAR/3 "Infarctions") OR ("Lateral" NEAR/3 "Medullary" NEAR/3 "Syndrome") OR ("Multi-Infarct" NEAR/3 "Dementia") OR ("transient" NEAR/3 "ischemic" NEAR/3 "attack") OR ("Transient" NEAR/3 "Brainstem" NEAR/3 "Ischemia") OR ("Transient" NEAR/3 "Brainstem" NEAR/3 "Ischemias") OR ("Transient" NEAR/3 "Cerebral" NEAR/3 "Ischemia") OR ("Transient" NEAR/3 "Cerebral" NEAR/3 "Ischemias") OR ("Transient" NEAR/3 "Ischemic" NEAR/3 "Attacks") OR ("Transient" NEAR/3 "Brainstem" NEAR/3 "Ischaemias") OR ("Transient" NEAR/3 "Brainstem" NEAR/3 "Ischaemia") OR ("Transient" NEAR/3 "Cerebral" NEAR/3 "Ischaemia") OR ("Transient" NEAR/3 "Cerebral" NEAR/3 "Ischaemias") OR ("Transient" NEAR/3 "Ischaemic" NEAR/3 "Attack") OR ("Transient" NEAR/3 "Ischaemic" NEAR/3 "Attacks")):ti,ab,kw AND ("Heart Atrium Appendage" OR "Atrial Appendage" OR "Atrial Appendages" OR "Atrium Appendage" OR "Atrium Appendages" OR "Ablation Therapy" OR "Left Atrial Appendage*" OR "LAA" OR "LAAs" OR "ablation" OR "ablat*" OR "pulmonary vein isolation" OR "PVI" OR (("Atrial" NEAR/3 "Appendage") OR ("Atrial" NEAR/3 "Appendages") OR ("Atrium" NEAR/3 "Appendage") OR ("Atrium" NEAR/3 "Appendages") OR ("pulmonary" NEAR/3 "vein" NEAR/3 "isolation"))):ti,ab,kw AND (("Mitral Annuloplasty" OR "Mitral Valve Prolapse" OR "Mitral Valve Annuloplasty" OR "Mitral Valve Prolapse" OR "Mitral Annuloplasty" OR "Mitral Prolapse" OR "Mitral valve" OR "Mitral valves" OR "Mitral valve*" OR "MVP" OR "MVr" OR (("Mitral" NEAR/3 "valve") OR ("Mitral" NEAR/3 "valves") OR ("Mitral" NEAR/3 "Annuloplasty") OR ("Mitral" NEAR/3 "Prolapse"))):ti,ab,kw **OR (("Heart Surgery" OR "Cardiac Surgery")**:ti **AND ("valve" OR "valves")**:ti,ab,kw**)**)) **OR** (("prophylactic" OR "prophyla*"):ti,ab,kw AND ("Heart Atrium Appendage" OR "Atrial Appendage" OR "Atrial Appendages" OR "Atrium Appendage" OR "Atrium Appendages" OR "Ablation Therapy" OR "Left Atrial Appendage*" OR "LAA" OR "LAAs" OR "ablation" OR "ablat*" OR "pulmonary vein isolation" OR "PVI" OR (("Atrial" NEAR/3 "Appendage") OR ("Atrial" NEAR/3 "Appendages") OR ("Atrium" NEAR/3 "Appendage") OR ("Atrium" NEAR/3 "Appendages") OR ("pulmonary" NEAR/3 "vein" NEAR/3 "isolation"))):ti,ab,kw AND (("Mitral Annuloplasty" OR "Mitral Valve Prolapse" OR "Mitral Valve Annuloplasty" OR "Mitral Valve Prolapse" OR "Mitral Annuloplasty" OR "Mitral Prolapse" OR "Mitral valve" OR "Mitral valves" OR "Mitral valve*" OR "MVP" OR "MVr" OR (("Mitral" NEAR/3 "valve") OR ("Mitral" NEAR/3 "valves") OR ("Mitral" NEAR/3 "Annuloplasty") OR ("Mitral" NEAR/3 "Prolapse"))):ti,ab,kw **OR ("Cardiac Surgery" OR "Cardiac Surgery")**:ti))**)**

(conference abstract OR meeting abstract OR conference proceeding OR conference proceedings):pt

**Emcare** <http://ovidsp.ovid.com/ovidweb.cgi?T=JS&NEWS=n&CSC=Y&PAGE=main&D=emcr>

**(**((exp *"Cerebrovascular Accident"/ OR exp *"Transient Ischemic Attack"/ OR "Stroke".ti,ab OR "Apoplexy".ti,ab OR "Brain Vascular Accident".ti,ab OR "Brain Vascular Accidents".ti,ab OR "Cerebrovascular Accident".ti,ab OR "Cerebrovascular Accident".ti,ab OR "Cerebrovascular Accidents".ti,ab OR "Cerebrovascular Accidents".ti,ab OR "CVA".ti,ab OR "CVAs".ti,ab OR "Brain Infarction".ti,ab OR "Brain Infarctions".ti,ab OR "Brain Stem Infarction".ti,ab OR "Brain Stem Infarctions".ti,ab OR "CADASIL".ti,ab OR "Cerebral Artery Infarction".ti,ab OR "Cerebral Artery Infarctions".ti,ab OR "Cerebral Infarction".ti,ab OR "Cerebral Infarctions".ti,ab OR "Lateral Medullary Syndrome".ti,ab OR "Multi-Infarct Dementia".ti,ab OR "transient ischemic attack".ti,ab OR "TIA".ti,ab OR "TIAs".ti,ab OR "Transient Brainstem Ischemia".ti,ab OR "Transient Cerebral Ischemia".ti,ab OR "Transient Cerebral Ischemias".ti,ab OR "Transient Ischemic Attacks".ti,ab OR "Transient Brainstem Ischaemia".ti,ab OR "Transient Cerebral Ischaemia".ti,ab OR "Transient Ischaemic Attack".ti,ab OR "Transient Ischaemic Attacks".ti,ab OR ("Brain" ADJ3 "Vascular" ADJ3 "Accident").ti,ab OR ("Brain" ADJ3 "Vascular" ADJ3 "Accidents").ti,ab OR ("Cerebrovascular" ADJ3 "Accident").ti,ab OR ("Cerebrovascular" ADJ3 "Accident").ti,ab OR ("Cerebrovascular" ADJ3 "Accidents").ti,ab OR ("Cerebrovascular" ADJ3 "Accidents").ti,ab OR ("Brain" ADJ3 "Infarction").ti,ab OR ("Brain" ADJ3 "Infarctions").ti,ab OR ("Brain" ADJ3 "Stem" ADJ3 "Infarction").ti,ab OR ("Brain" ADJ3 "Stem" ADJ3 "Infarctions").ti,ab OR ("Cerebral" ADJ3 "Artery" ADJ3 "Infarction").ti,ab OR ("Cerebral" ADJ3 "Artery" ADJ3 "Infarctions").ti,ab OR ("Cerebral" ADJ3 "Infarction").ti,ab OR ("Cerebral" ADJ3 "Infarctions").ti,ab OR ("Lateral" ADJ3 "Medullary" ADJ3 "Syndrome").ti,ab OR ("Multi-Infarct" ADJ3 "Dementia").ti,ab OR ("transient" ADJ3 "ischemic" ADJ3 "attack").ti,ab OR ("Transient" ADJ3 "Brainstem" ADJ3 "Ischemia").ti,ab OR ("Transient" ADJ3 "Brainstem" ADJ3 "Ischemias").ti,ab OR ("Transient" ADJ3 "Cerebral" ADJ3 "Ischemia").ti,ab OR ("Transient" ADJ3 "Cerebral" ADJ3 "Ischemias").ti,ab OR ("Transient" ADJ3 "Ischemic" ADJ3 "Attacks").ti,ab OR ("Transient" ADJ3 "Brainstem" ADJ3 "Ischaemias").ti,ab OR ("Transient" ADJ3 "Brainstem" ADJ3 "Ischaemia").ti,ab OR ("Transient" ADJ3 "Cerebral" ADJ3 "Ischaemia").ti,ab OR ("Transient" ADJ3 "Cerebral" ADJ3 "Ischaemias").ti,ab OR ("Transient" ADJ3 "Ischaemic" ADJ3 "Attack").ti,ab OR ("Transient" ADJ3 "Ischaemic" ADJ3 "Attacks").ti,ab) AND (exp *"Heart Atrium Appendage"/ OR "Atrial Appendage".ti,ab OR "Atrial Appendages".ti,ab OR "Atrium Appendage".ti,ab OR "Atrium Appendages".ti,ab OR exp *"Ablation Therapy"/ OR "Left Atrial Appendage*".ti,ab OR "LAA".ti,ab OR "LAAs".ti,ab OR "ablation".ti,ab OR "ablat*".ti,ab OR "pulmonary vein isolation".ti,ab OR "PVI".ti,ab OR (("Atrial" ADJ3 "Appendage") OR ("Atrial" ADJ3 "Appendages") OR ("Atrium" ADJ3 "Appendage") OR ("Atrium" ADJ3 "Appendages") OR ("pulmonary" ADJ3 "vein" ADJ3 "isolation")).ti,ab) AND (exp *"Mitral Annuloplasty"/ OR exp *"Mitral Valve Prolapse"/ OR "Mitral Valve Annuloplasty".ti,ab OR "Mitral Valve Prolapse".ti,ab OR "Mitral Annuloplasty".ti,ab OR "Mitral Prolapse".ti,ab OR "Mitral valve".ti,ab OR "Mitral valves".ti,ab OR "Mitral valve*".ti,ab OR "MVP".ti,ab OR "MVr".ti,ab OR (("Mitral" ADJ3 "valve") OR ("Mitral" ADJ3 "valves") OR ("Mitral" ADJ3 "Annuloplasty") OR ("Mitral" ADJ3 "Prolapse")).ti,ab **OR ((*"Heart Surgery"/ OR "Cardiac Surgery".ti) AND ("valve".ti,ab OR "valves".ti,ab))**)) **OR** (("prophylactic".ti,ab OR "prophyla*".ti,ab) AND (exp *"Heart Atrium Appendage"/ OR "Atrial Appendage".ti,ab OR "Atrial Appendages".ti,ab OR "Atrium Appendage".ti,ab OR "Atrium Appendages".ti,ab OR exp *"Ablation Therapy"/ OR "Left Atrial Appendage*".ti,ab OR "LAA".ti,ab OR "LAAs".ti,ab OR "ablation".ti,ab OR "ablat*".ti,ab OR "pulmonary vein isolation".ti,ab OR "PVI".ti,ab OR (("Atrial" ADJ3 "Appendage") OR ("Atrial" ADJ3 "Appendages") OR ("Atrium" ADJ3 "Appendage") OR ("Atrium" ADJ3 "Appendages") OR ("pulmonary" ADJ3 "vein" ADJ3 "isolation")).ti,ab) AND (exp *"Mitral Annuloplasty"/ OR exp *"Mitral Valve Prolapse"/ OR "Mitral Valve Annuloplasty".ti,ab OR "Mitral Valve Prolapse".ti,ab OR "Mitral Annuloplasty".ti,ab OR "Mitral Prolapse".ti,ab OR "Mitral valve".ti,ab OR "Mitral valves".ti,ab OR "Mitral valve*".ti,ab OR "MVP".ti,ab OR "MVr".ti,ab OR (("Mitral" ADJ3 "valve") OR ("Mitral" ADJ3 "valves") OR ("Mitral" ADJ3 "Annuloplasty") OR ("Mitral" ADJ3 "Prolapse")).ti,ab **OR *"Cardiac Surgery"/ OR "Cardiac Surgery".ti**))**)** NOT (("Case Report"/ OR "case report".ti OR ("case" AND ("report" OR "reports")).jx OR exp "Review"/ OR "review".ti) NOT ("Clinical Study"/ OR exp "Clinical Trial"/ OR "trial".ti OR "RCT".ti))

**Supplementary Material 2: Risk of bias (RoB 2.0)**


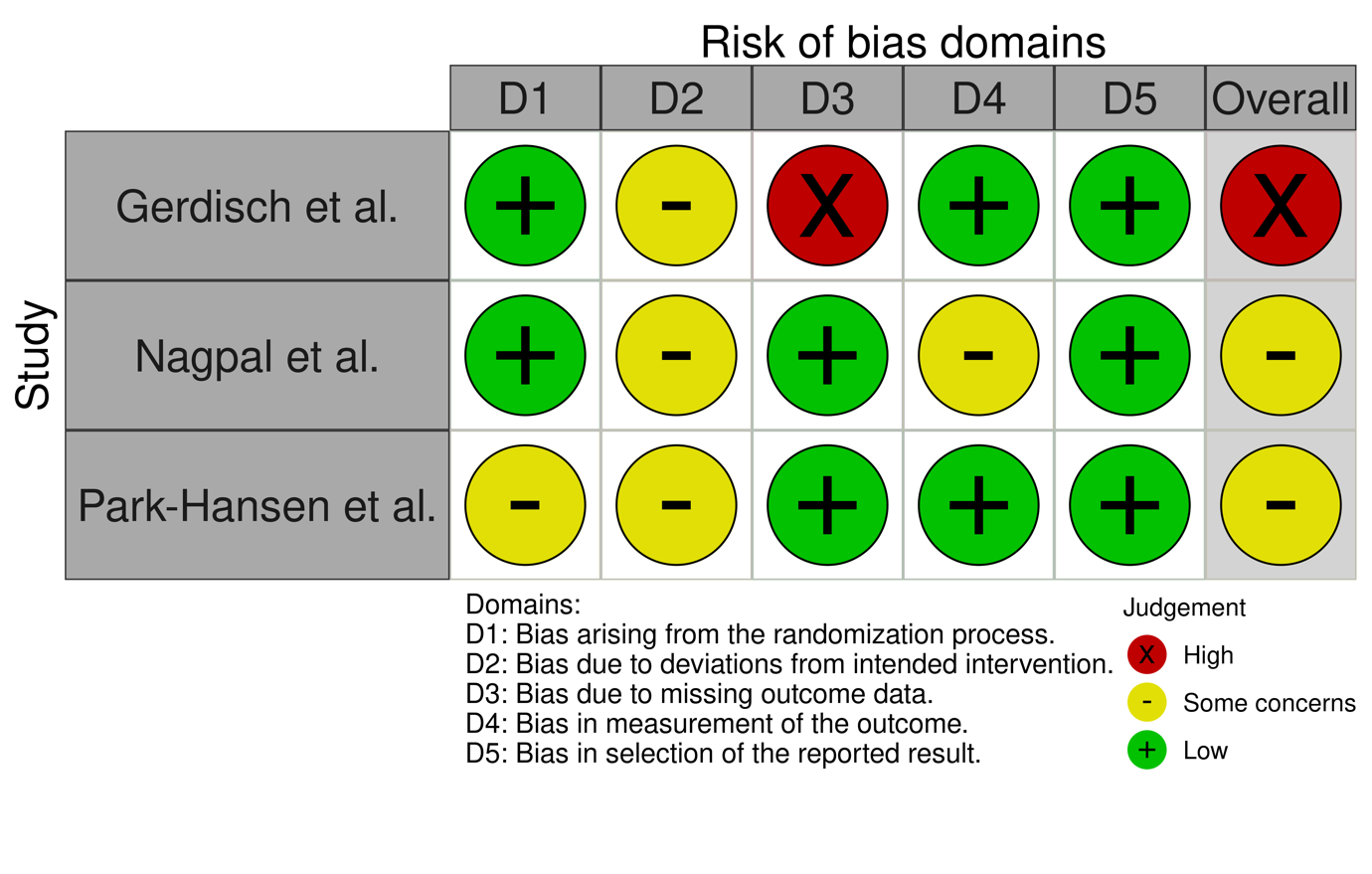


Risk of bias plot was generated using McGuinness, LA, Higgins, JPT. Risk-of-bias VISualization (robvis): An R package and Shiny web app for visualizing risk-of-bias assessments. Res Syn Meth. 2020; 1- 7. <https://doi.org/10.1002/jrsm.1411>

Gerdisch (RoB2)

| **Risk of bias arising from the randomization process** | **Low risk of bias.** Allocation sequence was random: Patients were randomized 2:1 by SAS version 9.4. Allocation sequence was probably concealed until participants were enrolled and assigned to interventions. Sealed envelopes were used. Baseline differences were negligible. The only characteristic that differed between the two groups was LVEF, but by a negligible amount.   \|  \| \| --- \| |
| --- | --- | --- |
| **Risk of bias due to deviations from the intended interventions** | **Moderate risk of bias.** ATLAS was an unblinded RCT: Participants and surgeons were aware of the assigned intervention during the trial. No information was given on cross-over between the treatment groups. Three patients in the LAAO did not undergo successful LAAO. |
| **Risk of bias due to missing outcome data** | **High risk of bias.** Patients who did not develop POAF prior to  hospital discharge were exited from the study at the 30-day  assessment. Thus, withdrawal from the study is related to the participant’s health status and on the true value (i.e. POAF yes/no) |
| **Risk of bias in measurement of the outcome** | **Low risk of bias**. Thromboembolic and hemorrhagic events, as well as deaths, were adjudicated by an independent physician who was not involved in the ATLAS trial. All patients were monitored per hospital standard of care for POAF after the index procedure. No difference in measurement means between the two groups. |
| **Risk of bias in selection of the reported result** | **Low risk of bias.** Adverse events were adjudicated for relatedness (i.e. analyzed) by investigators and an independent physician, probably in accordance to the protocol. |

Nagpal (RoB2)

| **Risk of bias arising from the randomization process** | **Low risk of bias**. The allocation sequence was random and done using computer-generation. This was concealed until participants were enrolled and assigned to interventions by sealed-envelope technique. No p-values were reported, however both groups were similar in terms of baseline characteristics. |
| --- | --- |
| **Risk of bias due to deviations from the intended interventions** | **Moderate risk of bias.** Of the 22 patients randomized to receive left atrial appendage exclusion, 18 actually did undergo successful exclusion. Reasons for not having the appendage excluded were a calcified left atrium in one patient, and surgical team oversight in the remaining three patients. All other treatments, including preoperative, intraoperative, and postoperative care were as per the  Hospital San Raffaele routine for all patients. |
| **Risk of bias due to missing outcome data** | **Low risk of bias.** Postoperative data is mainly based on the outcomes during hospitalization |
| **Risk of bias in measurement of the outcome** | **Moderate risk of bias**. Method of measuring was probably appropriate. No information on blinding of outcome assessors. Assessment could probably not be influenced by knowledge of intervention received. |
| **Risk of bias in selection of the reported result** | **Low risk of bias.** Data reporting adheres to the intention-to-treat principle |

Park-Hansen (RoB2)

| **Risk of bias arising from the randomization process** | **Moderate risk of bias.** Patients were randomized 1:1 by computer-generated randomization. Allocation sequence was probably  concealed until participants were enrolled and assigned to interventions. Although no p-values are given, imbalance in one or more key prognostic factors (i.e. pre-op AF, prior stroke, diabetes and age) seem statistically significantly different). |
| --- | --- |
| **Risk of bias due to deviations from the intended interventions** | **Moderate risk of bias.** Participants and surgeons were aware of the assigned intervention during the trial. There were deviations from the intended intervention but substantial cross-over was expected and addressed by compensatory randomization so it would not likely have affected the outcome |
| **Risk of bias due to missing outcome data** | **Low risk of bias.** Five patients (<5%) were lost to follow-up for the primary endpoint |
| **Risk of bias in measurement of the outcome** | **Low risk of bias.** Brain MRI’s were assessed by radiologists that were blinded to randomization. No difference in measurement were reported between the intervention groups. |
| **Risk of bias in selection of the reported result** | **Low risk of bias**. All outcome analyses were performed as intention to treat and per protocol. |

**Supplementary Material 2 (continued): Risk of bias (ROBINS-I)**


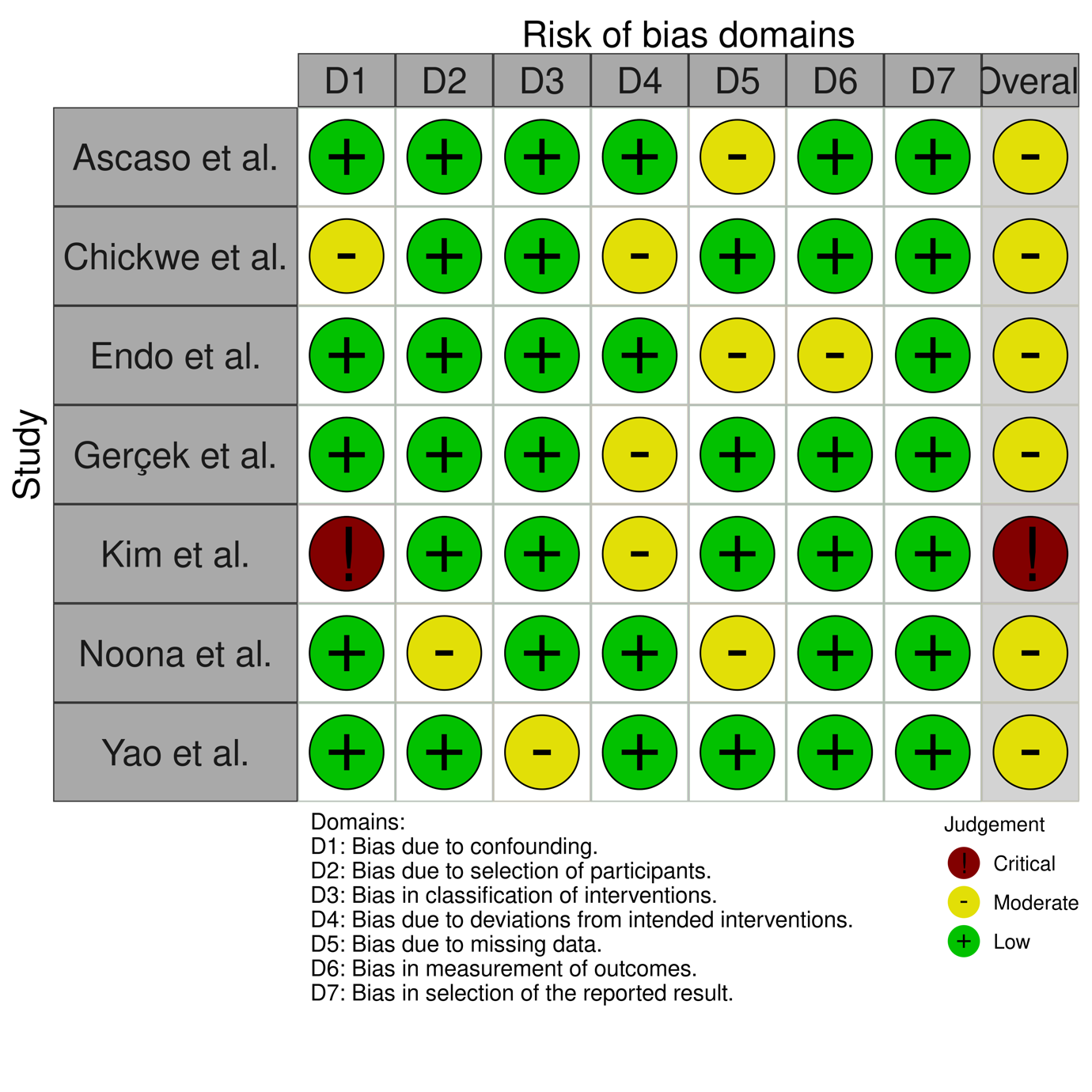


Risk of bias plot was generated using McGuinness, LA, Higgins, JPT. Risk-of-bias VISualization (robvis): An R package and Shiny web app for visualizing risk-of-bias assessments. Res Syn Meth. 2020; 1- 7. https://doi.org/10.1002/jrsm.1411

Ascaso (ROBINS-1)

| **Bias due to confounding** | **Low risk of bias.** Patients differed significantly from each other at baseline (in age, smoking, COPD, LVEF). Gender was not reported. All known important confounding domains were appropriately measured and controlled for: after PSM, SMD was low in all confounding domains between the two arms.  *and*  Reliability and validity of measurement of important domains were sufficient, such that we do not expect serious residual confounding. We used the PSM groups for our analysis. |
| --- | --- |
| **Bias in selection of participants into the study** | **Low risk of bias.** The study included consecutive patients from January 2000 through December 2019 operated by 1 surgeon. |
| **Bias in classification of interventions** | **Low risk of bias.** LAAO was closed with sutures. |
| **Bias due to deviations from intended interventions** | **Low risk of bias.** Interventions were carried out per ITT. |
| **Bias due to missing data** | **Moderate risk of bias.** Few of the covariates had missing values: 9 with missing age and 4 with missing ejection fraction. Thus, the data were reasonably complete. Furthermore, the analysis addressed the missing data by considering a mean amputation for the missing values in matching which might lead to bias. |
| **Bias in measurement of outcomes** | **Low risk of bias.** Outcome assessment was the same in both groups and unrelated to the intervention status. Furthermore, outcome measures such as POAF and stroke involve negligible assessor judgment. |
| **Bias in selection of the reported result** | **Low risk of bias.** All reported results correspond to all intended outcomes, analyses and subcohorts. |

Chikwe (ROBINS-1)

| **Bias due to confounding** | **Moderate of bias.** Confounding expected: patients differed significantly from each other at baseline (in age and urgency of the procedure). Cox regression was used to adjust for differences  in patient characteristics.  A separate sensitivity analysis was conducted that excluded patients undergoing concomitant cryomaze procedures. |
| --- | --- |
| **Bias in selection of participants into the study** | **Low risk of bias.** The study included consecutive patients from 2005 through 2020 undergoing robotic MVP operated by 5 surgeons at Cedars-Sinai Medical Center. |
| **Bias in classification of interventions** | **Low risk of bias.** |
| **Bias due to deviations from intended interventions** | **Moderate risk of bias.** Surgical practice rergarding LAAO changed in 2014: from selective to routine LAA closure: expectations of a difference between intervention and comparator from the surgeon’s perspective caused this deviation |
| **Bias due to missing data** | **Low risk of bias.** Outcome data were available for all participants. |
| **Bias in measurement of outcomes** | **Low risk of bias.** Measurements of the outcome were not carried out by the research team |
| **Bias in selection of the reported result** | **Low risk of bias.** |

Endo (ROBINS-1)

| **Bias due to confounding** | **Low risk of bias**. Patients from the original cohorts differed significantly from each other at baseline. However, all known important confounding domains were appropriately measured and controlled for: after IP weighting, SMD was low in all confounding domains between the two arms.  *and*  Reliability and validity of measurement of important domains were sufficient, such that we do not expect serious residual confounding. We used the PSM groups for our analysis. |
| --- | --- |
| **Bias in selection of participants into the study** | **Low risk of bias.** The study included consecutive patients from 2002 through 2020 undergoing cardiac surgery from 1 single center. |
| **Bias in classification of interventions** | **Low risk of bias.** |
| **Bias due to deviations from intended interventions** | **Low risk of bias.** All patients underwent enhanced CT postoperatively and intraoperative TEE to confirm complete closure of the LAA. |
| **Bias due to missing data** | **Moderate risk of bias.** Several patients were lost to follow-up due to non-compliance with a hospital visit or relocation to other areas. |
| **Bias in measurement of outcomes** | **Moderate risk of bias.** Follow-up was performed by unblinded physicians. In case patients could not visit the hospital, telephone contact would follow to ask the patients about their condition and symptoms of thromboembolic events. |
| **Bias in selection of the reported result** | **Low risk of bias.** All comparisons were probably based on the intention-to-treat principle |

Gerçek (ROBINS-1)

| **Bias due to confounding** | **Low risk of bias**. Patients from the original cohorts differed significantly from each other at baseline. However, all known important confounding domains were appropriately measured and controlled for: after PSM matching, SMD was low in all confounding domains between the two arms.  *and*  Reliability and validity of measurement of important domains were sufficient, such that we do not expect serious residual confounding. We used the PSM groups for our analysis. |
| --- | --- |
| **Bias in selection of participants into the study** | **Low risk of bias.** The study included consecutive patients from 2014 through 2016 undergoing cardiac surgery from 1 medical center. |
| **Bias in classification of interventions** | **Low risk of bias.** |
| **Bias due to deviations from intended interventions** | **Moderate risk of bias.** Decision making for an LAA amputation was based on a CHA₂DS₂-VASc Score of 2 or more and it was based on the surgeon’s perioperative judgement of safe execution. |
| **Bias due to missing data** | **Low risk of bias.** |
| **Bias in measurement of outcomes** | **Low risk of bias.** Patients’ medical records were examined and patients were probably monitored per hospital standard of care after the index procedure. No difference in measurement means between the two groups. |
| **Bias in selection of the reported result** | **Low risk of bias.** All comparisons were probably based on the intention-to-treat principle, no difference in means of reporting of results. |

Kim (ROBINS-1)

| **Bias due to confounding** | **High risk of bias**. Confounding expected: patients differed significantly from each other at baseline (CHF, HTN, AF). All known important confounding domains were appropriately measured and controlled for: after PSM, all confounding domains between the two arms were corrected for. Pre-operative AF status is not reported, thus leading to a high risk of bias. |
| --- | --- |
| **Bias in selection of participants into the study** | **Low risk of bias.** The study included consecutive patients from 2001 through 2010 undergoing cardiac surgery by 1 surgeon. |
| **Bias in classification of interventions** | **Low risk of bias.** |
| **Bias due to deviations from intended interventions** | **Moderate risk of bias.** In mid-2003, after the observation of a significant number of postoperative AF-related CVAs, there was a change in this surgeon’s (N.B.) standard practice to include exclusion, and later excision, of the LAA. |
| **Bias due to missing data** | **Low risk of bias.** |
| **Bias in measurement of outcomes** | **Low risk of bias.** Patients’ medical records were examined through postoperative day 30 for the incidence of postoperative AF and CVA. All data were obtained from the patients’ hospital and office medical records, and the hospital’s Society of Thoracic Surgeons cardiac database. All patients were probably monitored per hospital standard of care for POAF after the index procedure. No difference in measurement means between the two groups. |
| **Bias in selection of the reported result** | **Low risk of bias.** All comparisons were probably based on the intention-to-treat principle |

Noona (ROBINS-1)

| **Bias due to confounding** | **Low risk of bias**. Patients from the original cohorts differed significantly from each other at baseline. However, all known important confounding domains were appropriately measured and controlled for: after PSM matching, SMD was low in all confounding domains between the two arms.  *and*  Reliability and validity of measurement of important domains were sufficient, such that we do not expect serious residual confounding. We used the PSM groups for our analysis. |
| --- | --- |
| **Bias in selection of participants into the study** | **Moderate risk of bias.** The study included all patients from 2017 through 2023 undergoing cardiac surgery by the Virginia Cardiac Services Quality Initiative (VCSQI) from 17 different centers. Only patients who received devide-based LAAO were included the LAAO group. |
| **Bias in classification of interventions** | **Low risk of bias.** The patients in the LAAO group underwent LAAO with the same device based technique. |
| **Bias due to deviations from intended interventions** | **Low risk of bias.** Any deviations from intended intervention reflected usual practice |
| **Bias due to missing data** | **Moderate risk of bias.** VCSQI records were examined for patient demographics, operative data and postoperative outcomes. However, no measure was described in case data was missing from the VCSQI. |
| **Bias in measurement of outcomes** | **Low risk of bias.** **Low risk of bias.** Patients’ medical records were examined and patients were probably monitored per hospital standard of care after the index procedure. No difference in measurement means between the two groups. |
| **Bias in selection of the reported result** | **Low risk of bias.** All comparisons were probably based on the intention-to-treat principle |

Yao (ROBINS-1)

| **Bias due to confounding** | **Low risk of bias.** Most important confounding domains were appropriately measured and controlled for in the subgroup analysis for patients without pre-operative atrial fibrillation: after PSM, no significant baseline differences existed between the two study groups. |
| --- | --- |
| **Bias in selection of participants into the study** | **Low risk of bias.** The study (probably consecutively) included patients from 2009-2017. |
| **Bias in classification of interventions** | **Moderate risk of bias.** Intervention groups were not clearly defined: the study was unable to distinguish between LAAO by excision or exclusion by sutures or stapling. |
| **Bias due to deviations from intended interventions** | **Low risk of bias.** Any deviations from intended intervention reflected usual practice. |
| **Bias due to missing data** | **Low risk of bias.** Data was probably reasonably complete and missing data was imputed. Also, missing values were balanced after matching on almost all patient characteristics, meaning that proportions for missing data was similar across intervention groups. |
| **Bias in measurement of outcomes** | **Low risk of bias.** The study relied on administrative data to ascertain outcomes, which could be subject to misclassification. However, the same method was used across the intervention groups. |
| **Bias in selection of the reported result** | **Low risk of bias.** All reported results seem to correspond to all intended outcomes, analyses and subcohorts. |

**Supplementary Material 3: Baseline characteristics**

| First author | Ascaso [10] | | Chickwe [11] | | Gerdisch [12] | | Kim [13] | | Nagpal [14] | |
| --- | --- | --- | --- | --- | --- | --- | --- | --- | --- | --- |
| Pre-operative | **LAAO** | **Control** | **LAAO** | **Control** | **LAAO** | **Control** | **LAAO** | **Control** | **LAAO** | **Control** |
| N | 267 | 267 | 431 | 333 | 376 | 186 | 631 | 631 | 22 | 21 |
| Age | 58.8 (median) | 59.2 (median) | 63 (median) | 57 (median) | 69.2 (mean) | 68.9 (mean) | 66.2 (mean) | 65.7 (mean) | 57.8 (mean) | 59.2 (mean) |
| Male | ND | ND | 68.2% (294/431) | 67.6% (225/333) | 69.9% (263/376) | 73.1% (136/186) | 68.1% (430/631) | 68.1% (430/631) | 50% (11/22) | 57.1% (12/21) |
| HTN | 34.5% (92/267) | 32.6% (87/267) | ND | ND | ND | ND | 74.5% (470/631) | 74.5% (470/631) | ND | ND |
| DM | 3.4% (9/267) | 3.0% (8/267) | 4.9% (21/431) | 5.4% (18/333) | ND | ND | 34.2% (216/631) | 34.2% (216/631) | 4.5% (1/22) | 0% |
| History of AF | 0% | 0% | 0% | 0% | 0% | 0% | ND | ND | 18.2 % (4/22) | 19.0% (4/21) |
| Prior thromboembolic complications | 4.9% (13/267) | 3.7% (10/267) | 0.7% (3/431) | 0.9% (3/333) | ND | ND | 4.9% (31/631) | 4.9% (31/631) | 0% | 4.8% (1/21) |
| CHA(2)DS(2)-VASc | ND | ND | 2 (median) | 1 (median) | 3.4 (mean) | 3.4 (mean) | 2.3 (mean) | 2.3 (mean) | ND | ND |
| Intra-operative |  |  |  |  |  |  |  |  |  |  |
| CABG | 0% | 0% | 0% | 0% | 82.2% (309/376) | 85.5% (159/186) | 92.4% (583/631) | 92.4% (583/631) | 0% | 0% |
| MV | 100% | 100% | 100% | 100% | 5.9% (22/376) | 4.3% (8/186) | ND | ND | 100% | 100% |

Supplementary Material 3: Baseline characteristics (continued)

| First author | Park-Hansen [15] | | Yao [16] | | Gerçek [17] | | Endo [18] | | Noona [19] | |
| --- | --- | --- | --- | --- | --- | --- | --- | --- | --- | --- |
| Pre-operative | **LAAO** | **Control** | **LAAO** | **Control** | **LAAO** | **Control** | **LAAO** | **Control** | **LAAO** | **Control** |
| N | 101 | 86 | 1076 | 1076 | 243 | 243 | 236.5 | 264.4 | 439 | 439 |
| Age | 67.6 (mean) | 69.3 (mean) | 64.3 (mean) | 64.3 (mean) | 69.1 (mean) | 69.6 (mean) | 64.0 (mean) | 64.0 (mean) | 67.0 (mean) | 67.0 (mean) |
| Male | 83.2 (84/101) | 87.2% (75/86) | 64.8% (697/1076) | 66.7% (718/1076) | 77.8% (189/243) | 80.2% (195/243) | 83.0% (196.3/236.5) | 83.7% (221.3/264.4) | 69.0% (303/439) | 68.1% (299/439) |
| HTN | 74.3% (75/101) | 69.8% (60/86) | ND | ND | 91.8% (223/243) | 93.0% (226/243) | 82.6% (203.9/236.5) | 83.2% (220.0/264.4) | 92.3% (405/439) | 91.1% (400/439) |
| DM | 30.7% (31/101) | 22.1% (19/86) | ND | ND | 43.2% (105/243) | 41.2% (100/243) | 72.3% (171.0/236.5) | 71.6% (189.3/264.4) | 59.5% (261/439) | 58.1% (255/439) |
| History of AF | 16.8% (18/101) | 12.8% (12/86) | 0% | 0% | 0% | 0% | 0% | 0% | 0% | 0% |
| Prior thromboembolic complications | 10.9% (11/101) | 17.4% (15/86) | 14.4% (155/1076) | 12.8% (138/1076) | ND | ND | 22.9% (54.2/236.5) | 22.5% (59.5/264.4) | 15.3% (67/439) | 14.1% (62/439) |
| CHA(2)DS(2)-VASc | 2.9 (mean) | 2.9 (mean) | Score ≥4: 25.4% (273/1076) | Score ≥4:  24.5% (264/1076) | 3.1 (mean) | 3.1 (mean) | ND | ND | 4.0 (mean) | 4.0 (mean) |
| Intra-operative |  |  |  |  |  |  |  |  |  |  |
| CABG | 49.5% (50/101) | 46.5% (40/86) | 33.6% (361/1076) | 34.0% (366/1076) | 100% | 100% | 100% | 100% | 100% | 100% |
| MV | 6.9% (7/101) | 4.7% (4/86) | ND | ND | 0% | 0% | 0% | 0% | 0% | 0% |
| Abbreviations: LAAO: left atrial appendage occlusion; PVI: pulmonary vein isolation; N: number of patients; HTN: hypertension; DM: diabetes mellitus; AF: atrial fibrillation; CABG: coronary artery bypass graft; MV: mitral valve surgery; ND: not determined | | | | | | | | | | |

**Supplementary Material 7: PRISMA 2020 checklist**

| **Section and Topic** | **Item #** | **Checklist item** | **Location where item is reported** |
| --- | --- | --- | --- |
| **TITLE** | | |  |
| Title | 1 | Identify the report as a systematic review. | 1 |
| **ABSTRACT** | | |  |
| Abstract | 2 | See the PRISMA 2020 for Abstracts checklist. | 2 |
| **INTRODUCTION** | | |  |
| Rationale | 3 | Describe the rationale for the review in the context of existing knowledge. | 3 |
| Objectives | 4 | Provide an explicit statement of the objective(s) or question(s) the review addresses. | 3 |
| **METHODS** | | |  |
| Eligibility criteria | 5 | Specify the inclusion and exclusion criteria for the review and how studies were grouped for the syntheses. | 4 |
| Information sources | 6 | Specify all databases, registers, websites, organisations, reference lists and other sources searched or consulted to identify studies. Specify the date when each source was last searched or consulted. | 4 |
| Search strategy | 7 | Present the full search strategies for all databases, registers and websites, including any filters and limits used. | SM1 |
| Selection process | 8 | Specify the methods used to decide whether a study met the inclusion criteria of the review, including how many reviewers screened each record and each report retrieved, whether they worked independently, and if applicable, details of automation tools used in the process. | 4 |
| Data collection process | 9 | Specify the methods used to collect data from reports, including how many reviewers collected data from each report, whether they worked independently, any processes for obtaining or confirming data from study investigators, and if applicable, details of automation tools used in the process. | 4-5 |
| Data items | 10a | List and define all outcomes for which data were sought. Specify whether all results that were compatible with each outcome domain in each study were sought (e.g. for all measures, time points, analyses), and if not, the methods used to decide which results to collect. | 5 |
|  | 10b | List and define all other variables for which data were sought (e.g. participant and intervention characteristics, funding sources). Describe any assumptions made about any missing or unclear information. | SM3 |
| Study risk of bias assessment | 11 | Specify the methods used to assess risk of bias in the included studies, including details of the tool(s) used, how many reviewers assessed each study and whether they worked independently, and if applicable, details of automation tools used in the process. | 5 |
| Effect measures | 12 | Specify for each outcome the effect measure(s) (e.g. risk ratio, mean difference) used in the synthesis or presentation of results. | Fig2-4 |
| Synthesis methods | 13a | Describe the processes used to decide which studies were eligible for each synthesis (e.g. tabulating the study intervention characteristics and comparing against the planned groups for each synthesis (item #5)). | 5-6 |
|  | 13b | Describe any methods required to prepare the data for presentation or synthesis, such as handling of missing summary statistics, or data conversions. | 5-6 |
|  | 13c | Describe any methods used to tabulate or visually display results of individual studies and syntheses. | 5-6 |
|  | 13d | Describe any methods used to synthesize results and provide a rationale for the choice(s). If meta-analysis was performed, describe the model(s), method(s) to identify the presence and extent of statistical heterogeneity, and software package(s) used. | 5-6 |
|  | 13e | Describe any methods used to explore possible causes of heterogeneity among study results (e.g. subgroup analysis, meta-regression). | N/A |
|  | 13f | Describe any sensitivity analyses conducted to assess robustness of the synthesized results. | N/A |
| Reporting bias assessment | 14 | Describe any methods used to assess risk of bias due to missing results in a synthesis (arising from reporting biases). | 5 |
| Certainty assessment | 15 | Describe any methods used to assess certainty (or confidence) in the body of evidence for an outcome. | 5 |
| **RESULTS** | | |  |
| Study selection | 16a | Describe the results of the search and selection process, from the number of records identified in the search to the number of studies included in the review, ideally using a flow diagram. | 6; Fig1 |
|  | 16b | Cite studies that might appear to meet the inclusion criteria, but which were excluded, and explain why they were excluded. | 6 |
| Study characteristics | 17 | Cite each included study and present its characteristics. | Tb1 |
| Risk of bias in studies | 18 | Present assessments of risk of bias for each included study. | 7; SM2 |
| Results of individual studies | 19 | For all outcomes, present, for each study: (a) summary statistics for each group (where appropriate) and (b) an effect estimate and its precision (e.g. confidence/credible interval), ideally using structured tables or plots. | Fig2-4 |
| Results of syntheses | 20a | For each synthesis, briefly summarise the characteristics and risk of bias among contributing studies. | SM2 |
|  | 20b | Present results of all statistical syntheses conducted. If meta-analysis was done, present for each the summary estimate and its precision (e.g. confidence/credible interval) and measures of statistical heterogeneity. If comparing groups, describe the direction of the effect. | Fig2-4 |
|  | 20c | Present results of all investigations of possible causes of heterogeneity among study results. | N/A |
|  | 20d | Present results of all sensitivity analyses conducted to assess the robustness of the synthesized results. | N/A |
| Reporting biases | 21 | Present assessments of risk of bias due to missing results (arising from reporting biases) for each synthesis assessed. | SM2 |
| Certainty of evidence | 22 | Present assessments of certainty (or confidence) in the body of evidence for each outcome assessed. | Fig2-4 |
| **DISCUSSION** | | |  |
| Discussion | 23a | Provide a general interpretation of the results in the context of other evidence. | 9-11 |
|  | 23b | Discuss any limitations of the evidence included in the review. | 11 |
|  | 23c | Discuss any limitations of the review processes used. | 11 |
|  | 23d | Discuss implications of the results for practice, policy, and future research. | 9-11 |
| **OTHER INFORMATION** | | |  |
| Registration and protocol | 24a | Provide registration information for the review, including register name and registration number, or state that the review was not registered. | N/A |
|  | 24b | Indicate where the review protocol can be accessed, or state that a protocol was not prepared. | N/A |
|  | 24c | Describe and explain any amendments to information provided at registration or in the protocol. | N/A |
| Support | 25 | Describe sources of financial or non-financial support for the review, and the role of the funders or sponsors in the review. | 13 |
| Competing interests | 26 | Declare any competing interests of review authors. | 13 |
| Availability of data, code and other materials | 27 | Report which of the following are publicly available and where they can be found: template data collection forms; data extracted from included studies; data used for all analyses; analytic code; any other materials used in the review. | 13 |
